# Automated classification of tropical shrub species: a hybrid of leaf shape and machine learning approach

**DOI:** 10.7717/peerj.3792

**Published:** 2017-09-12

**Authors:** Miraemiliana Murat, Siow-Wee Chang, Arpah Abu, Hwa Jen Yap, Kien-Thai Yong

**Affiliations:** 1 Bioinformatics Programme, Institute of Biological Sciences, Faculty of Science, University of Malaya, Kuala Lumpur, Malaysia; 2 Department of Mechanical Engineering, Faculty of Engineering, University of Malaya, Kuala Lumpur, Malaysia; 3 Ecology and Biodiversity Programme, Institute of Biological Sciences, Faculty of Science, University of Malaya, Kuala Lumpur, Malaysia

**Keywords:** Tropical shrubs, Shape descriptor, Classification, Machine learning, Feature selection

## Abstract

Plants play a crucial role in foodstuff, medicine, industry, and environmental protection. The skill of recognising plants is very important in some applications, including conservation of endangered species and rehabilitation of lands after mining activities. However, it is a difficult task to identify plant species because it requires specialized knowledge. Developing an automated classification system for plant species is necessary and valuable since it can help specialists as well as the public in identifying plant species easily. Shape descriptors were applied on the myDAUN dataset that contains 45 tropical shrub species collected from the University of Malaya (UM), Malaysia. Based on literature review, this is the first study in the development of tropical shrub species image dataset and classification using a hybrid of leaf shape and machine learning approach. Four types of shape descriptors were used in this study namely morphological shape descriptors (MSD), Histogram of Oriented Gradients (HOG), Hu invariant moments (Hu) and Zernike moments (ZM). Single descriptor, as well as the combination of hybrid descriptors were tested and compared. The tropical shrub species are classified using six different classifiers, which are artificial neural network (ANN), random forest (RF), support vector machine (SVM), k-nearest neighbour (k-NN), linear discriminant analysis (LDA) and directed acyclic graph multiclass least squares twin support vector machine (DAG MLSTSVM). In addition, three types of feature selection methods were tested in the myDAUN dataset, Relief, Correlation-based feature selection (CFS) and Pearson’s coefficient correlation (PCC). The well-known Flavia dataset and Swedish Leaf dataset were used as the validation dataset on the proposed methods. The results showed that the hybrid of all descriptors of ANN outperformed the other classifiers with an average classification accuracy of 98.23% for the myDAUN dataset, 95.25% for the Flavia dataset and 99.89% for the Swedish Leaf dataset. In addition, the Relief feature selection method achieved the highest classification accuracy of 98.13% after 80 (or 60%) of the original features were reduced, from 133 to 53 descriptors in the myDAUN dataset with the reduction in computational time. Subsequently, the hybridisation of four descriptors gave the best results compared to others. It is proven that the combination MSD and HOG were good enough for tropical shrubs species classification. Hu and ZM descriptors also improved the accuracy in tropical shrubs species classification in terms of invariant to translation, rotation and scale. ANN outperformed the others for tropical shrub species classification in this study. Feature selection methods can be used in the classification of tropical shrub species, as the comparable results could be obtained with the reduced descriptors and reduced in computational time and cost.

## Introduction

Plants form a fundamental part of life on earth in providing oxygen, food, medicine and fuel. Plants also play an important role in environmental protection (Tilman et al., 2002). However, the increasing anthropogenic pressure on the natural environments has driven many of the native plant species toward the verge of extinction (Hore et al., 1997; Mata-Montero & Carranza-Rojas, 2016; Menges, 1998). The resulting ecological crisis has brought many serious consequences including flash floods, regional climate changes, desertification and so on (Geertsema, Highland & Vaugeouis, 2009; Wiens, 2016). In general, people nowadays have better understanding about the importance and urgency to conserve plant resources. In order to conserve the correct plant species, it is important for the general public to be able to recognise and identify the many plant species, toward the protection of important local plant species (Corlett, 2016).

There are about 500,000 plant species that are present in the world and it is difficult for any botanist or researcher to know more than a tiny fraction of the total number of known species (Fu & Chi, 2006). Even though humans try to recognize a plant species based on botanical and biological characteristics, species identification actually requires vast knowledge and in depth training in botany and plant systematics. Thus, manual recognition is always time consuming and inefficient (Wu et al., 2007); even professional botanists take plenty of time to master plant species identification (Rademaker, 2000). Therefore, many researchers are focusing on developing a user-friendly plant species identification mechanism or an automated system that could assist the recognition process (Kumar et al., 2012; Wang et al., 2008).

Plants are generally classified based on their leaf and flower characteristics where leaves are virtually 2D in shape but flowers are 3D (Viscosi & Cardini, 2011). Therefore, it is challenging to analyse the structure and the shape of the flowers since they have complex 3D structures (Kellogg, 2015). Furthermore, leaves can be easily found and collected at all seasons, while flowers are only available during the blooming season (Chaki, Parekh & Bhattacharya, 2015; Tomar & Agarwal, 2016). The leaves can be recognized based on their shapes, textures and colours using shape descriptors, which are the most commonly used plant species classification system (Oncevay-Marcos et al., 2015).

The aims of this study are to classify tropical shrub species based on leaf shape descriptors and to compare different feature selection methods with classification tool. Based on literature reviews, this is the first study in the development of tropical shrub species image dataset and classification using a hybrid of leaf shape and machine learning approach. The classification of tropical shrub species was conducted using either single or a combination of four different shape descriptors which are, morphological shape descriptors (MSD), Histogram of Oriented Gradients (HOG), Hu invariant moments and Zernike moments. Three features selection methods which were Relief, Correlation-based feature selection (CFS) and Pearson’s correlation coefficient (PCC) were tested on the proposed tropical shrub species dataset, and the selected descriptors were classified using artificial neural network (ANN), random forest (RF), support vector machine (SVM), k-nearest neighbour (k-NN), linear discriminant analysis (LDA) and directed acyclic graph multiclass least squares twin support vector machine (DAG MLSTSVM).

## Background Study

Plant identification based on leaf characteristics were suggested by many researchers. Most of the time, the shape, colour, and leaf texture were referred. Several features, including Fourier descriptors, Zernike moments, Legendre moments and Chebyshev moments, were compared in an attempt to recognize wooden tree species based on their leaves (Suk, Flusser & Novotný, 2013). Fourier descriptors slightly outperform the other tested features, recorded an 85% accuracy, and are the most convenient features in the experiment. Another group of researchers (Shanwen & YouQian, 2010) acquired Zernike moments and HOG method as shape descriptors, and reported 84.66% accuracy for Zernike moments and 92.67% accuracy for HOG. Aakif & Khan (2015) proposed an algorithm that incorporated morphological features, Fourier descriptors and shape defining features. The research was conducted on both Flavia and ICL datasets and both reported 96% accuracy. Ahmed, Khan & Asif (2016) proposed an approach that involved mainly the extracted features based of leaf shape. The feature set was based on 12 geometrical features, five vein features and Fourier descriptors. The accuracy was 87.4% when tested on Flavia dataset.

A system for identifying plants using shape, veins, colour and textures features that combined with Zernike moments was proposed by Kulkarni et al. (2013). The researchers used Zernike moments from order 2 to 10 along with five shape features, five colour features, 16 texture features and three vein features. Radial basis probabilistic Neural Network (RBPNN) was used as a classifier and achieved an accuracy of 93.82%. Pornpanomchai et al. (2011) developed a system that could recognize some Thai herb leaves. The system extracted 13 features from the leaf image and achieved 93.29% accuracy with k-nearest neighbours (k-NN) as a classifier. Sharma & Gupta (2015) presents an automated recognition system for the plants leaf image by using multilayer feed forward neural network and back propagation algorithm. A total of 12 features were extracted and this experiment performed with an accuracy of 91.13%. Du et al. (2006) proposed a computer-aided plant species identification method named as CAPSI, which was based on plant leaf images using shape-matching technique. Six methods were implemented which were Fourier descriptors, Hu invariant moment, contour moment, curvature scale space, geometrical features and Modified dynamic programming (MDP). The experiment recorded an accuracy up to 92% using k-NN classifier. Kadir et al. (2012) proposed a method using Zernike moments, which involved a combination of three features namely, geometric, colour moments and gray–level co-occurrence matrices (GLCM). The experiment showed that Zernike moments work better when they were combined with other features in leaf recognition systems. By using the proposed system with Euclidean distance, City block distance and Probabilistic Neural Network (PNN), the accuracy were 94.69%, 93.75% and 93.44% respectively.

## Material and Methods

### Sampling sites and data collection

**Figure 1:**
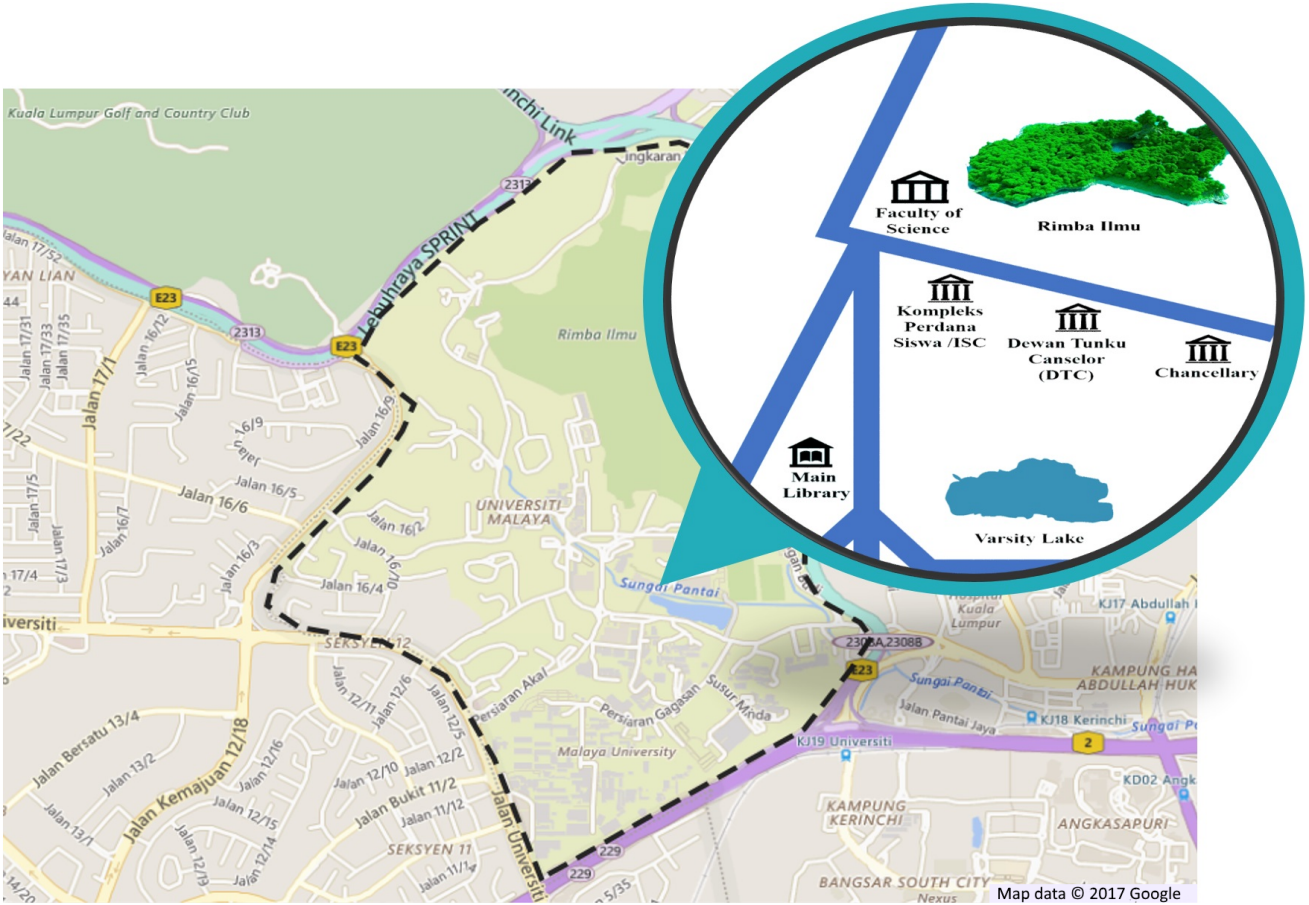
Location of sampling area in the University of Malaya (UM), Kuala Lumpur, Malaysia.

This dataset is named as myDAUN, in which ‘my’ represents Malaysia and ‘DAUN’ means leaf in the Malay language. The images in the myDAUN dataset were sampled from the campus of University of Malaya (UM), Kuala Lumpur, Malaysia. UM is Malaysia’s oldest university and is situated in the southwest of Kuala Lumpur, the capital of Malaysia. There is a botanical garden, Rimba Ilmu situated in the UM campus with over 1,600 plant species that emphasises the flora of the Malaysian and Indonesian region (University of Malaya, 1991). The myDAUN dataset was initially focused only on shrub species that are commonly seen by the public. Four main locations in UM with more variety of tropical shrubs were chosen and the sampling took place in these locations. The four locations were Faculty of Science, Dewan Tunku Canselor (Tunku Canselor Hall), Varsity Lake and Main Library as shown in Fig. 1.

The species of tropical shrub were identified and selected with the help of botanists. Since shrubs have a variety of species and cultivar, thus the advice from the professional botanists and the staff from the botanical garden were crucial and valuable. In this study, 45 species of common tropical shrubs were selected and 30 leaf samples were collected for each species. Hence, there were 1350 images of tropical shrub leaf images in total. Table 1 shows the selected species in the myDAUN database.

### Proposed framework

**Figure 2:**
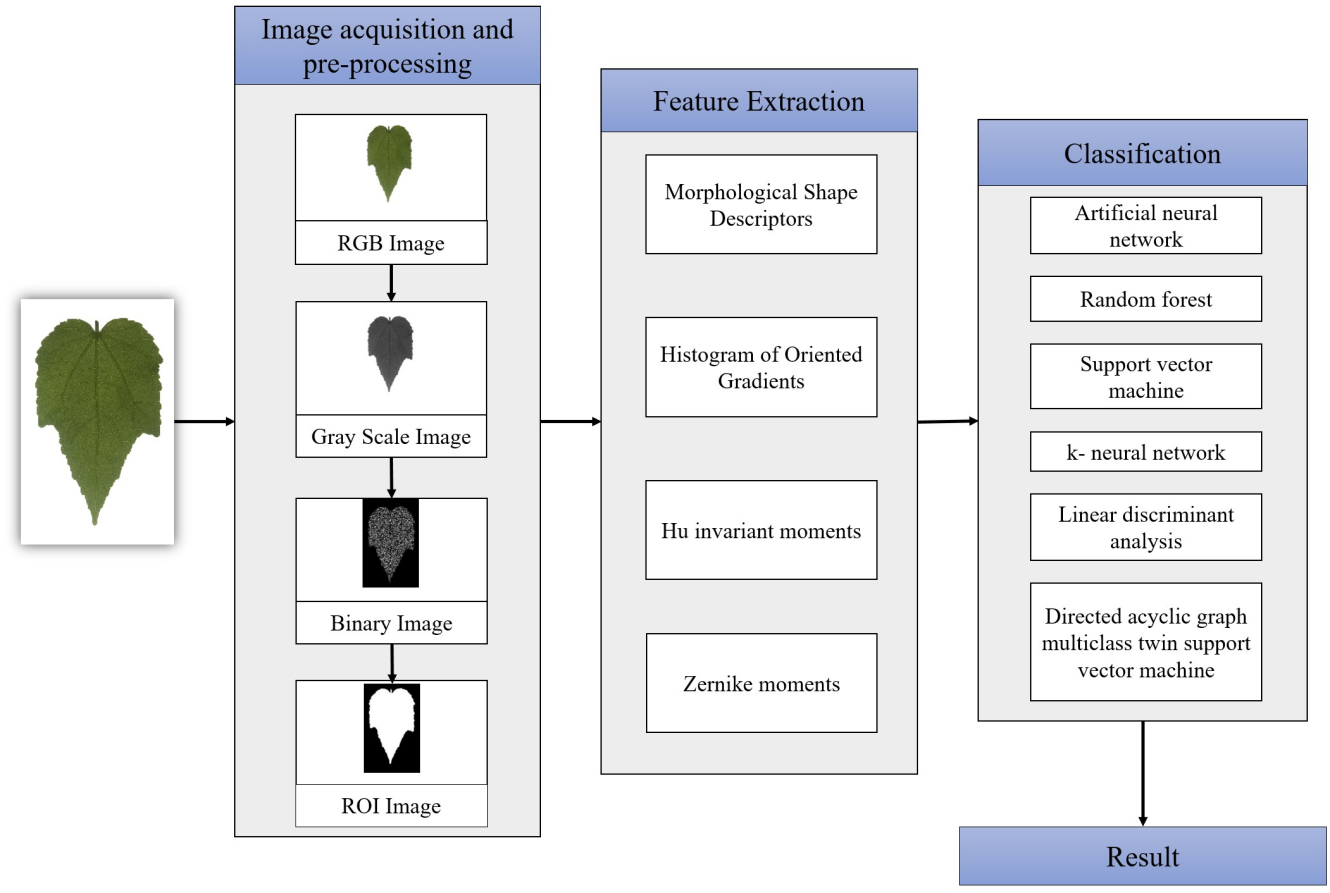
Flowchart of the proposed methodology.

The implementation of the tropical shrub species classification system is shown in Fig. 2. There are four main steps; image acquisition, image pre-processing, features extraction and the output of the classification process. Matlab R2015b was used to develop and test the proposed method.

### Image acquisition

**Figure 3:**
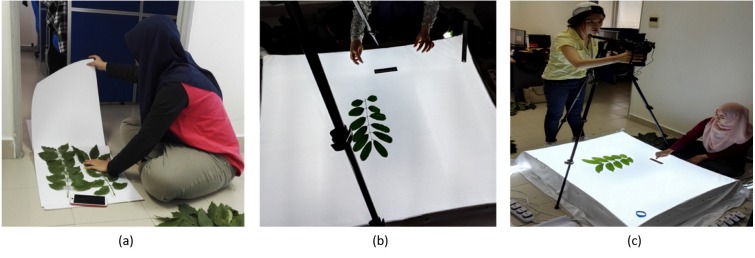
Experimental setup. (A) Leaf compression, (B) Background setup, (C) Overview of experimental setup.

The myDAUN image dataset was collected and compiled in the laboratory in UM. Firstly, leaf samples that could represent the existing population were identified and the leaf samples from different parts of the shrub and size were plucked and collected. The standard criteria is to select a leaf that has not ruptured, is abnormal or damaged. Secateurs was used for a clean cut of the stem. Thirty samples of leaf were collected for each species. Each of the samples was labelled and the tags were attached securely onto the samples. Next, the collected samples were brought back to the laboratory for image acquisition. The samples were compressed and flattened using newspapers, and the leaf stalks were removed. In order to obtain a quality leaf photo, the light boxes were designed and two sizes of light boxes were used which were 37 cm × 59 cm × 13.5 cm for small-sized leaves and 93 cm × 111 cm  × 13.5 cm for bigger-sized leaves. The setup of image acquisition step is shown in Fig. 3.

**Figure 4:**
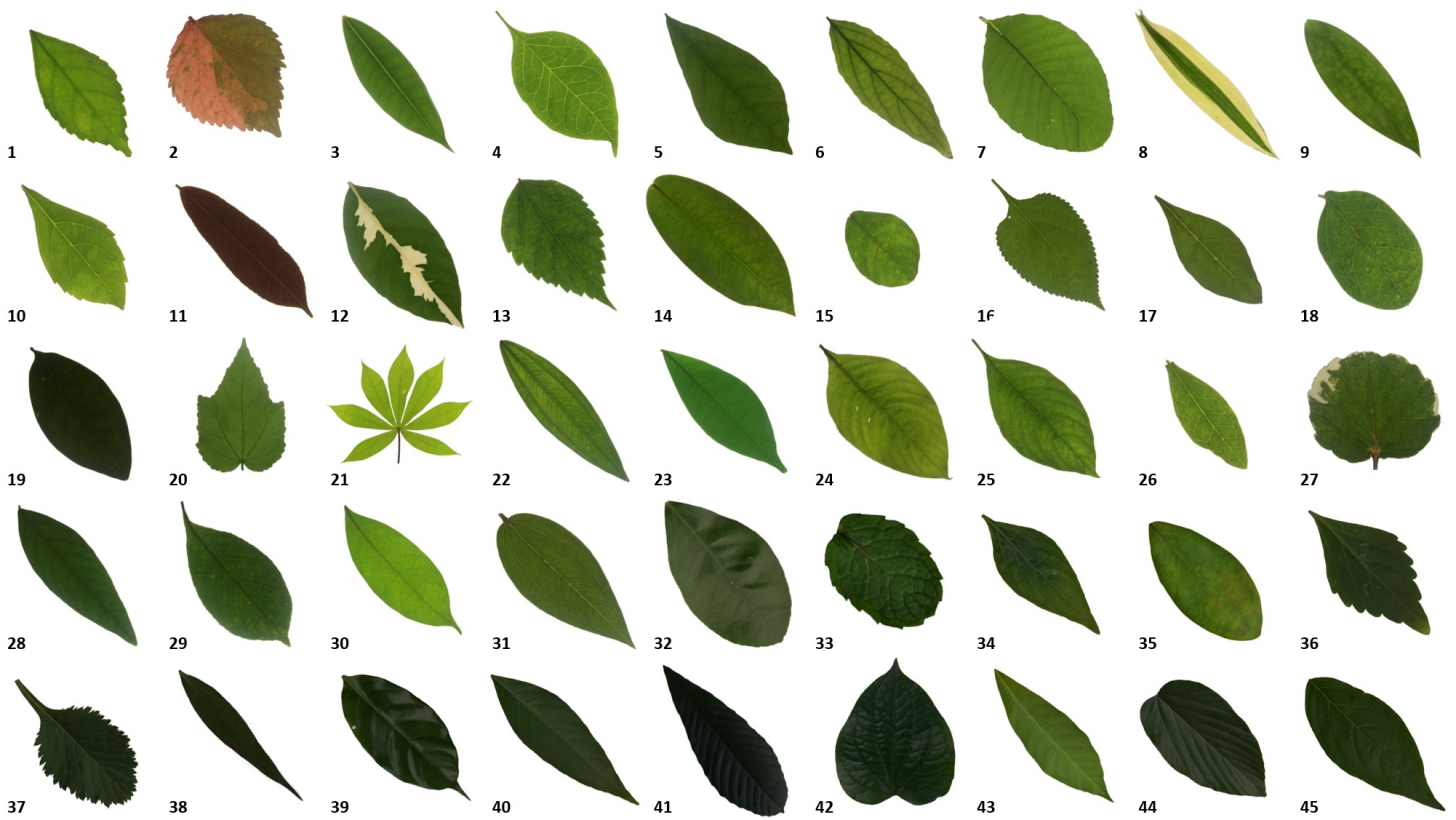
Samples of the leaf images in the myDAUN dataset.

The captured images are taken in the same standard with uniform background. The image of the leaf samples is captured on the front side, from a distance of 55 cm from the camera lens using Nikon D750 DSLR camera with AF-S Nikkor 24–120 mm F4G ED VR lens, with a resolution of 6,014 × 4,016 pixels and stored as 32-bits RGB colour Tagged Image File Format (tiff). Adobe Photoshop CC was used to enhance the image quality and to eliminate the illumination and contrast problem, which would affect the process of object segmentation. Figure 4 shows the samples of all tropical shrub species in the myDAUN dataset.

### Image pre-processing

**Figure 5:**
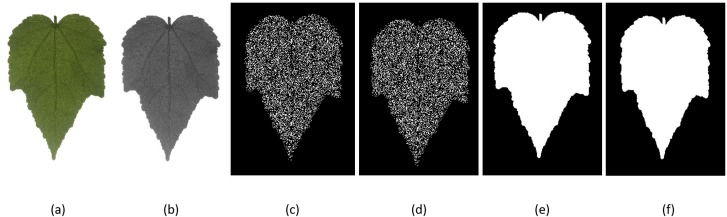
Image pre-processing. (A) original image, (B) grayscale image, (C) detected edge, (D) binary image, (E) filled binary image, (F) ROI image.

The main objectives of image pre-processing are to identify the main object, which is the leaf shape, and get rid of all other unrelated information. Before morphological descriptors were extracted, the region of interest (ROI) of the leaf must be obtained. Firstly, the RGB image or original image is converted to grayscale image. Next, Canny edge detection method is applied to the leaf grey scale images. The image with detected edge is then converted to a binary image and the shape of object is obtained after filling the holes in the binary image. To obtain the desired shape of leaf in the image, the small particles in the surrounding are removed and the ROI was obtained through the segmentation process. The steps for image pre-processing is shown in Fig. 5.

**Table 1:** List of tropical shrub species in the myDAUN dataset.

Location	Label	Scientific name	Common name
Faculty of Science	1	*Acalypa siamensis*	Tea Leaves
	2	*Acalypha wilkesiana*	Copperleaf
	5	*Brunfelsia calycina*	Yesterday, Today and Tomorrow
	6	*Clinacanthus nutans*	Sabah Snake Grass
	7	*Dillenia suffruticosa*	Yellow Simpoh
	8	*Dracaena surculosa*	Japanese Bamboo
	9	*Dracaena reflexa*	Song of India
	12	*Graptophyllum pictum*	Caricature
	15	*Lagerstroemia indica*	Crepe Myrtle
	16	*Lantana camara*	Lantana
	17	*Lawsonia inermia*	Henna
	19	*Magnolia figo*	Banana Shrub
	20	*Malvaviscus arboreus*	Sleepy Mallow
	22	*Melastoma malabathricum*	Sesenduk
	27	*Polyscias balfouriana*	Dinner-plate Aralia
	28	*Sauropus androgyrus*	Star Gooseberry
	29	*Strobilanthes crispa*	Bayam Karang
	30	*Tabernaemontana divaricata*	Ceylon Jasmine
	31	*Tibouchina urvilleana*	Glory Bush
	32	*Citrus microcarpa*	China orange
	33	*Mentha piperita*	Peppermint
	34	*Andrographis paniculata*	King of bitters
	35	*Rhodomyrtus tomentosa*	Downy rose myrtle
	36	*Orthosiphon aristatus*	Cat’s whiskers
	37	*Centratherum punctatum*	Lark daisy
	38	*Polygonum minus*	Laksa leaf
	40	*Justicia gendarussa*	Gendarusa
	41	*Tetracera scandens*	Stone leaf
	42	*Piper sarmentosum*	Wild pepper
	44	*Flemingia strobilifera*	Wild hops
	45	*Cananga odorata*	Ylang- ylang
Tunku Canselor Hall	10	*Duranta erecta*	Golden Dew-Drop
	11	*Excoecaria cochinchinensis*	Chinese Croton
	14	*Ixora javanica*	Jungle Geranium
	23	*Murraya paniculata*	Kemuning
	24	*Mussaenda erythrophylla*	Red Flag Bush
	25	*Mussaenda philippica*	White Mussaenda
	26	*Phyllanthus myrtifolius*	Ceylon Myrtle
	43	*Rauvolfia serpentine*	Indian snakefoot
Varsity Lake	3	*Allamanda cathartica*	Golden Trumpet
	4	*Bougainvillea spectabilis*	Great Bougainvillea
	13	*Hibiscus rosa-sinensis*	Chinese Hibiscus
Main Library	18	*Loropetalum chinense*	Chinese Fringe-flower
	21	*Manihot esculenta*	Manihot
	39	*Tabernaemontana coronaria*	Crepe jasmine

### Feature extraction

The ROIs obtained from the image pre-processing step are used as input in the feature extraction steps. Four types of shape representations are applied and tested, which were morphological shape descriptors (MSD), Histogram of Oriented Gradients (HOG), Hu invariant moments and Zernike moments.

### Morphological shape descriptors (MSD)

Five basic shape descriptors commonly used for leaf analysis were used in this study, namely diameter, major axis length, minor axis length, area, and perimeter. Based on these basic shape descriptors, 15 morphological descriptors are computed as shown in Table 2.

### Histogram of oriented gradients (HOG)

The Histogram of Oriented Gradients (HOG) are descriptors used in image processing for object detection and it is the local statistic of the orientations of the image gradients around key points (Dalal & Triggs, 2005; Xiao et al., 2010). HOG descriptors method determines occurrences of gradient orientation in localized portions of an image or ROI. This technique is alike to scale-invariant feature transformation descriptors, edge orientation histograms and shape context. Gradient computation, *G* and gradient orientation, *θ* are computed using Eqs. (1) and (2) respectively.

(1) \begin{eqnarray*}{|}G{|}& =& \sqrt{{G}_{x}^{2}+{G}_{y}^{2}};\end{eqnarray*}

(2) \begin{eqnarray*}\theta & =& arctan \frac{{G}_{x}}{{G}_{y}} ;\end{eqnarray*}

where *G*_*x*_ is gradient in *X* direction and *G*_*y*_ is gradient in *Y* direction.

Each pixel within a cell casts a weighted vote for an orientation based histogram based on the gradient magnitude, *G*, and gradient orientation, *θ*. One histogram was counted for each cell based on the number of bins orientation binning. After that, the image was split into a number of cells. A cell can be represented as a region like a square with a predefined size in pixels. Each block has 3 × 3 cells; for each cell, the histogram of the gradient is obtained by splitting votes into bins for each orientation. The normalization was executed among a group of cells called a block, and a normalization factor was calculated over the block. All the histograms within this block were normalized and link together in single feature vector. The normalized vector, *V* can be performed by

(3) \begin{eqnarray*}V= \frac{{V}_{K}}{{\mathop{\parallel {V}_{K}\parallel }\nolimits }_{2}^{2}+{\varepsilon }^{2}} ;\end{eqnarray*}

**Table 2:** Basic geometrical and morphological descriptors.

Diameter	Major axis length	Minor axis length	Area	Perimeter
Aspect ratio	Form factor	Rectangularity	Solidity	Eccentricity
Narrow factor	Convex area	Irrectangularity	Entirety	Equivalent diameter
Perimeter ratio of major axis length and minor axis length	Perimeter of convexity	Perimeter of area	Perimeter ratio of diameter	Perimeter ratio of major axis length

where *V*_*K*_ is the vector for combined histogram and ε is a small constant.

The histogram of all the blocks accumulated into a whole HOG descriptor were processed. In this study, the number of bins, K was set to 9, whereas the block size was 3 × 3 cells. Thus, there were 81-dimensional vector for each of leaf image based on the computation of 3 × 3 × 9.

### Hu invariant moments

The moment invariant was first introduced by Hu (1962). Hu defined seven invariant moments computed from central moments through order up to three and two-dimensional that are invariant under object translation, scaling and rotation. Hence, a set of seven invariant moments can be derived from the normalized central moments as stated in Eq. (4).

\begin{eqnarray*}Hu1& =& {\eta }_{20}+{\eta }_{02}; \end{eqnarray*}

\begin{eqnarray*}Hu2& =& { \left( {\eta }_{20}-{\eta }_{02} \right) }^{2}+4{\eta }_{11}^{2}; \end{eqnarray*}

\begin{eqnarray*}Hu3& =& { \left( {\eta }_{30}-3{\eta }_{12} \right) }^{2}+{ \left( {\eta }_{03}-3{\eta }_{21} \right) }^{2}; \end{eqnarray*}

(4) \begin{eqnarray*}Hu4& =& { \left( {\eta }_{30}+3{\eta }_{12} \right) }^{2}+{ \left( {\eta }_{03}+{\eta }_{21} \right) }^{2};\end{eqnarray*}

\begin{eqnarray*}Hu5& =& \left( {\eta }_{30}-3{\eta }_{12} \right) \left( {\eta }_{30}+{\eta }_{12} \right) \left[ { \left( {\eta }_{30}+{\eta }_{12} \right) }^{2}-3{ \left( {\eta }_{03}+{\eta }_{21} \right) }^{2} \right] \nonumber\\\displaystyle & +& \left( 3{\eta }_{21}-{\eta }_{03} \right) \left( {\eta }_{21}+{\eta }_{03} \right) \left[ 3{ \left( {\eta }_{30}+{\eta }_{12} \right) }^{2}-{ \left( {\eta }_{03}+{\eta }_{21} \right) }^{2} \right] ; \end{eqnarray*}

\begin{eqnarray*}Hu6& =& \left( {\eta }_{20}-{\eta }_{02} \right) \left[ { \left( {\eta }_{30}+{\eta }_{12} \right) }^{2}-{ \left( {\eta }_{03}+{\eta }_{21} \right) }^{2} \right] +4{\eta }_{11} \left( {\eta }_{30}+{\eta }_{12} \right) \left( {\eta }_{03}+{\eta }_{21} \right) ; \end{eqnarray*}

\begin{eqnarray*}Hu7& =& \left( 3{\eta }_{21}-{\eta }_{03} \right) \left( {\eta }_{30}+{\eta }_{12} \right) \left[ { \left( {\eta }_{30}+{\eta }_{12} \right) }^{2}-3{ \left( {\eta }_{03}+{\eta }_{21} \right) }^{2} \right] \nonumber\\\displaystyle & +& \left( 3{\eta }_{21}-{\eta }_{30} \right) \left( {\eta }_{21}+{\eta }_{03} \right) \left[ 3{ \left( {\eta }_{30}+{\eta }_{12} \right) }^{2}-{ \left( {\eta }_{03}+{\eta }_{21} \right) }^{2} \right] ; \end{eqnarray*}

### Zernike moments

Zernike moments was firstly introduced by Teague (1980). In order to compute Zernike moments, three steps are required, namely computation of radial polynomials, Zernike basis functions, and Zernike moments. The approach to obtain Zernike moments from an input image starts with the computation of Zernike radial polynomials (Hwang & Kim, 2006). Zernike moments are based on Zernike polynomials that are orthogonal to the circle *x*^2^ + *y*^2^ = 1. The form of these polynomials is formulated in Eq. (5)

(5) \begin{eqnarray*}{K}_{ab} \left( x,y \right) ={R}_{ab} \left( r \right) \exp \nolimits \left( jb\theta \right) ;\end{eqnarray*}

where *a* is a non-negative integer, *b* is positive or negative integer satisfying constrains *a* − |*b*| = even and |*b*| ≤ *a*. *r* is the radius of (*x*, *y*) to the centroid where $r=\sqrt{{x}^{2}+{y}^{2}}$, *θ* is the angle between *r* and *x*-axis where $\theta =ta{n}^{-1} \frac{y}{x} ,j=\sqrt{-1}$. *R*_*ab*_ is the radial polynomial defined as

(6) \begin{eqnarray*}{R}_{ab} \left( r \right) =\sum _{s=0}^{ \left( a- \left\vert b \right\vert \right) /2}{ \left( -1 \right) }^{s} \frac{ \left( a-s \right) {!}}{s{!} \left[ \frac{a+ \left\vert b \right\vert }{2} -s \right] {!} \left[ \frac{a- \left\vert b \right\vert }{2} -s \right] {!}} {r}^{a-2s};\end{eqnarray*}

The Zernike moments for order *a* and *b* repetition of continued function *f(x,y),* if *f(x,y)* is a digital image, is defined below:

(7) \begin{eqnarray*}{Z}_{ab}= \frac{a+1}{\pi } \sum _{x}\sum _{y}f \left( x,y \right) {K}_{ab}^{\ast } \left( r,\theta \right) ;\end{eqnarray*}

In this case, *K*^∗^ is the complex conjugate, while *K*_*ab*_ is the Zernike basis functions order *a* with *b* repetitions, where ${K}_{ab} \left( x,y \right) ={K}_{ab} \left( r,\theta \right) ={R}_{ab} \left( r \right) \exp \left( jb\theta \right) $.

These descriptors need to be normalized before classification. The normalized Zernike moments can be calculated using

(8) \begin{eqnarray*}{Z}_{ab}^{{^{\prime}}}= \frac{{Z}_{ab}}{{m}_{00}} ;\end{eqnarray*}

where *m*_00_ is spatial moment order (0,0) that indicates the area of a leaf.

The Zernike moments with order *a* counting from 0 to 8 as the descriptors were selected and 25 descriptors of Zernike moment were obtained.

### Feature selection

Feature selection is a process of identifying and removing the irrelevant and redundant features to describe the target concept. Feature selection reduced the dimensionality of the data and allowed learning algorithms to operate faster and more effectively. In this study, three feature selection methods are proposed, which are Relief, Correlation-based feature selection (CFS), and Pearson’s correlation coefficient (PCC). Relief is a distance based filter model that distinguish classes based on how well a feature can separate classes. CFS is a simple filter algorithm that ranks feature subset according to a correlation based heuristic evolution function (Hall, 1999). The PCC is a statistical method to analyse the relationship between features and decide which features are selected to train classifier.

### Classification

Six classifiers were tested and applied, which are artificial neural network (ANN), random forest (RF), support vector machine (SVM), k-nearest neighbour (k-NN), linear discriminant analysis (LDA) and directed acyclic graph multiclass least squares twin support vector machine (DAG MLSTSVM). All of the classification algorithms were tested using a random sampling approach. The myDAUN dataset was randomly divided into 80% for training and 20% for testing. This process was repeated 10 times and the average of 10 runs was taken as the final result.

### Artificial neural network (ANN)

**Figure 6:**
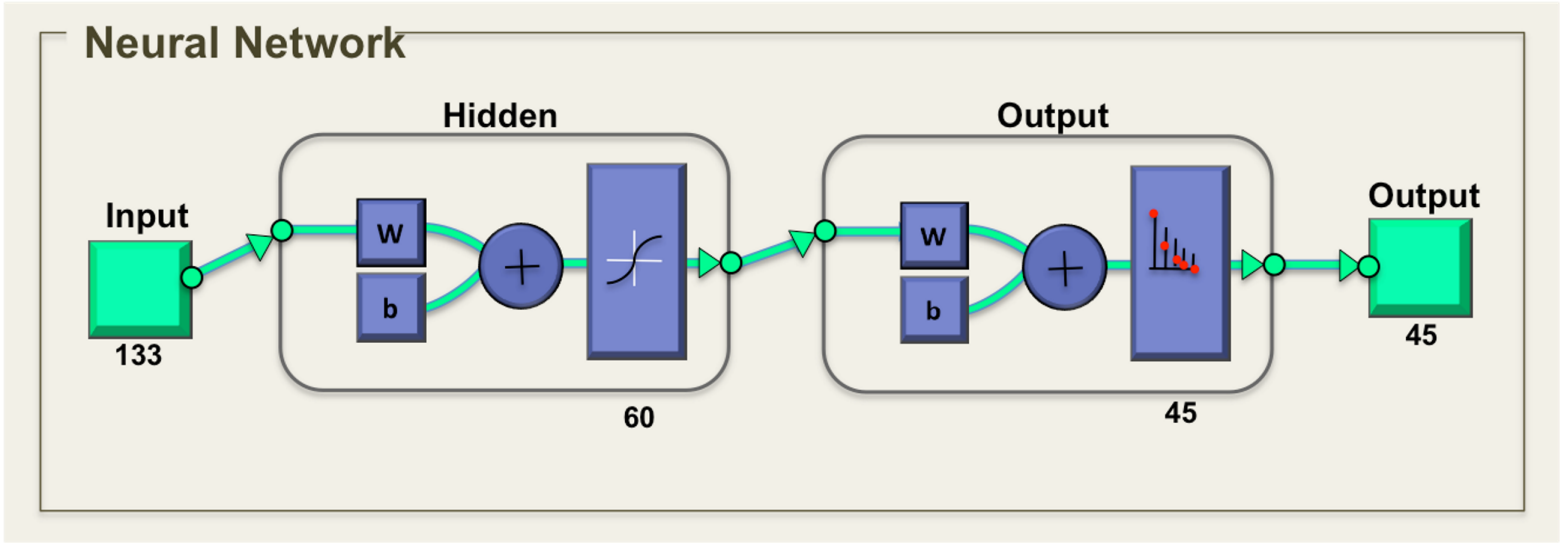
Neural network for tropical shrub species classification.

Artificial neural network (ANN) is used as the classification tool. ANN is a biologically inspired program designed to stimulate the system in which the human brain processes information. The general neural network consists of three layers, which are input layer, hidden layer, and output layer. The ANN is composed of a set of neurons that is interconnected with each other. The total of 133 descriptors that was obtained, which includes 20 descriptors from MSD descriptor, 81 descriptors from HOG descriptors, seven descriptors from Hu moments and 25 descriptors from Zernike moments. The number of neurons in the hidden layer was experimentally selected from the error set by comparing with the general training of the ANN. The number of output neurons was presented by the number of species classified, which in this case, are 45 classes. The networks were two-layer feed forward with 133 input nodes and 45 output nodes as shown in Fig. 6.

### Random forest (RF)

Random forest (RF) is based on the classification tree approach (Breiman, 2001). Predictions of multiple classification trees are aggregated for a dataset and each tree in the forest is grown using bootstrap samples. At prediction time, trees in the forest use their votes for target class and classification results are taken from each tree. The forest selected the class that achieved the most votes among the separate trees. RF can give an estimate of important input variables in the classification and it runs efficiently on large dataset with high accuracy. However, RF has some constraints on computing time and memory.

### Support vector machine (SVM)

Support vector machine (SVM) (Cortes & Vapnik, 1995) is a classification method that maps the input data to a high dimensional feature space through some nonlinear transformation that separated by optimal hyperplane, which maximizes the gap of positive samples and negative samples. SVM used the kernel function to transform the input data into a higher dimensional space and optimal hyperplane is constructed with maximum margin. The classification involved 45 classes in this study, therefore SVM classifier was trained using the one versus all approach. In this approach, every class was trained with test cases of that class as positive and all other as negative.

### k-nearest neighbour (k-NN)

k-nearest neighbour (k-NN) is a non-parametric classification algorithm that classifies unknown samples based on their k-nearest neighbour (Singh, Haddon & Markou, 2001). The most frequent class among these k neighbours is selected as the class of this sample. A challenge of k-NN is to determine a fit value of k, which is based on error rates. In these experiments, k-NN classifier with *k* = 1, 2, 3, 4, 5 were simulated and the case of *k* = 1 showed the best classification result.

### Linear discriminant analysis (LDA)

Linear discriminant analysis (LDA) is also known as the Fisher discriminant analysis (FDA) (Alpaydin, 2014). LDA proposed to find a linear combination of features, which characterizes or separates different classes. LDA maximizes the ratio of between class variance to within class variance, thus achieving maximum discrimination. LDA contains weights for each feature separately for every class that allows it to ignore features that have no significant meaning for some classes.

### Directed acyclic graph multiclass least squares twin support vector machine (DAG MLSTSVM)

Directed acyclic graph multiclass least squares twin support vector machine (DAG MLSTSVM) is a learning framework using directed acyclic graph that choose and reconstruct classifiers in “one-versus-one” algorithm. DAG MLSTSVM can solve a linear equation problem instead of using quadratic programming in the multiclass classification, which can lead to higher computational costs (Tomar & Agarwal, 2016).

## Result

The myDAUN dataset was tested using various sets of descriptors which were single and hybrid of two, three and four descriptors. The average classification accuracy using single descriptors are shown in Table 3. MSD, HOG and Zernike moments achieved the best classification with ANN accuracy for the single descriptor, which were 96.39%, 95.82% and 91.79%, respectively, whereas the highest accuracy of Hu invariant moments descriptor obtained 85.76% by using the DAG MLSTSVM classifier.

**Table 3:** Classification accuracy for single descriptor.

	^a^Average accuracy (%)					
Descriptor	ANN	RF	SVM	k-NN	LDA	DAG MLSTSVM
MSD	**96.39**	92.58	79.78	91.96	82.80	95.78
HOG	**95.82**	91.58	84.53	90.40	79.76	95.40
Hu invariant moments	82.27	83.36	32.74	82.99	37.65	**85.76**
Zernike moments	**91.79**	87.85	59.34	87.75	56.40	90.54

**Notes.**

a average accuracy = average of 10 runs.

Table 4 shows the average classification accuracy using hybridisation of descriptors. By comparing the classification accuracy in Table 3 and Table 4, it is clear that the hybridisation of descriptors improved the classification accuracy. The best classification accuracy for hybrid of two descriptors was demonstrated with ANN using MSD and HOG descriptor obtaining accuracy of 97.49% compared to other hybrid of two descriptors using RF, SVM, k-NN, LDA and DAG MLSTSVM. The greatest improvement was observed for the Hu invariant moments descriptor after hybridised with MSD, HOG and Zernike moments descriptors by using SVM and LDA classifiers with accuracy increased to 68.31% and 87.72% from 32.74% and 37.65%, respectively. The hybridisation of two and three descriptors achieved almost comparable results. The best classification accuracy result for the hybrid of three descriptors using MSD, HOG, and Zernike moments descriptors were 97.63% in ANN and 97.05% in DAG MLSTSVM, whereas in RF, k-NN and LDA the best accuracy results for hybrids of three descriptors were using MSD, HOG and Hu moments with 93.62%, 92.42% and 90.09% respectively. The average classification accuracy using the hybrid of all descriptors improved the classification accuracy of tropical shrub species and produced the best results in all classifiers. The best classification accuracy was 98.23% when combined with four descriptors of MSD, HOG, Hu invariant moments and Zernike moments using ANN.

**Table 4:** Classification accuracy of hybrid descriptors.

Methods	Descriptor	^a^Average accuracy (%)					
		ANN	RF	SVM	k-NN	LDA	DAG MLSTSVM
Hybrid of two descriptors	MSD + HOG	**97.49**	93.45	91.01	92.03	89.56	96.94
	MSD + Hu invariant moments	96.67	92.84	81.99	92.35	85.14	95.96
	MSD + Zernike moments	96.60	92.86	84.61	91.92	84.31	96.25
	HOG + Hu invariant moments	96.24	92.39	87.72	91.07	83.47	95.88
	HOG + Zernike moments	93.70	92.58	89.93	91.47	85.58	93.52
	Hu moments + Zernike moments	93.67	90.07	73.45	89.35	68.31	92.65
Hybrid of three descriptors	MSD + HOG + Hu invariant moments	97.59	93.62	91.78	92.42	90.09	96.99
	MSD + HOG + Zernike moments	**97.63**	93.52	92.06	92.17	89.72	97.05
	MSD + Hu moments + Zernike moments	96.64	93.24	87.93	92.10	86.12	96.32
	HOG + Hu moments + Zernike moments	97.06	93.37	91.29	91.56	87.23	96.70
Hybrid of all descriptors	MSD + HOG + Hu invariant moments + Zernike moments	**98.23**	93.83	92.74	92.60	90.86	97.72

**Notes.**

a average of 10 runs.

Table 5 shows the classification results using three feature selection methods. Relief, CFS and PCC were employed. The classification accuracy achieved using feature selection methods is between 96.98% to 98.13%. The Relief method with reduced feature of 60%(53 descriptors) achieved the best accuracy of 98.13%, which was comparable with the result without feature selection method of 98.23% (133 descriptors).

**Table 5:** Classification accuracy for the selected feature selection methods.

Descriptors	Descriptors reduced (%)	^a^Relief (%)	^a^CFS (%)	^a^PCC (%)
Hybrid of all descriptors	50	97.69	97.79	97.33
	60	98.13	96.98	97.10
	70	97.64	97.10	97.15
	None	**98.23%**		

**Notes.**

a average of 10 runs.

The computational time for feature extraction are reported in Table 6. The total computational time for feature extraction of all descriptors was 2,263.40 min, whereas the computational time for feature extraction using Relief method with 60% of reduced feature was 1,033.23 min. These results showed that by using feature selection of Relief, the total computational time had reduced by 1,230.17 min of 54.35% from the original full model.

**Table 6:** Running time for features extraction.

Descriptors	Time for all features extraction (min)	Time for features extraction with Relief (min)
MSD	84.01	61.04
HOG	334.39	334.39
HU	225.00	189.55
Zernike	1620.00	748.25
Total	2263.40	1033.23

### Validation using Flavia dataset and Swedish Leaf dataset

The purpose of validation is to test on the viability of using hybridisation of four descriptors on the classification of plant species in other datasets. To validate the proposed methods, the Flavia dataset and Swedish Leaf dataset were used. Flavia and Swedish Leaf dataset are currently the most popular benchmark datasets used by researchers to compare and evaluate methods across studies. The dataset of Flavia consists of 32 species with 50 samples for each species. Whereas, Swedish Leaf dataset consists of 15 species with 75 samples per species. The validation applied the same settings as in the myDAUN dataset with the ratio of training and testing data set to 80:20 and used ANN as the classifier. The results obtained from both datasets were comparable with the myDAUN dataset, where the combination of MSD, HOG, Hu moments and Zernike moments are descriptors obtained the best accuracy.

Table 7 shows the average accuracy for various sets of descriptors in Flavia dataset and Swedish Leaf dataset. For single descriptor method, MSD and HOG descriptor achieved more than 93% accuracy in the Flavia dataset and more than 98% in the Swedish Leaf dataset. Hu invariant moments obtained the lowest accuracy in the classification of plant species, which was only 80.46% in the Flavia dataset and 95.20% in the Swedish Leaf dataset. The highest accuracy for the hybrid of two, three and four descriptors increased slightly for both dataset. The combination of all descriptors improved the classification accuracy and produced the best result for classification of plant species in the Flavia and Swedish Leaf datasets. The results achieved in the Flavia and Swedish Leaf datasets were comparable to those achieved in the myDAUN dataset.

**Table 7:** Classification results of the Flavia dataset and the Swedish Leaf dataset.

Methods	Descriptor	^a^Average accuracy (%)	
		**Flavia**	**Swedish leaf**
Single descriptor	MSD	93.30	98.65
	HOG	93.49	99.15
	Hu invariant moments	80.46	95.20
	Zernike moments	83.22	95.95
Hybrid of two descriptors	MSD + HOG	95.04	99.54
	MSD + Hu invariant moments	93.12	98.37
	MSD + Zernike moments	93.41	99.16
	HOG + Hu invariant moments	93.55	99.24
	HOG + Zernike moments	93.87	99.54
	Hu invariant moments + Zernike moments	88.47	98.01
Hybrid of three descriptors	MSD + HOG + Hu invariant moments	94.01	99.43
	MSD + HOG + Zernike moments	95.14	99.64
	MSD + Hu invariant moments + Zernike moments	93.67	99.16
	HOG + Hu invariant moments + Zernike moments	94.08	99.52
Hybrid of all descriptors	MSD + HOG + Hu invariant moments + Zernike moments	**95.25**	**99.89**

**Notes.**

a average of 10 runs.

## Discussion

From the results in Table 3, the single descriptor that obtained the best accuracy was MSD with 96.39% using ANN classifier. Twenty descriptors of MSD were sufficient to give meaningful analysis for shape descriptor. However, there are risks to describe the leaf shape using only the MSD descriptors, even though they seem good enough to classify a small set of test images. Additionally, many single value descriptors in MSD were extremely correlated with each other, thus making the task of selecting sufficiently independent descriptors more difficult (Cope et al., 2012).

The HOG descriptors performed better than Zernike moments and Hu invariant moments because it induced robust shape descriptors. The result of single descriptor of HOG showed that most of the tropical shrub species with different leaf shapes were correctly identified. However, several cases were not well recognized. This is because HOG is computed over image, therefore the local information might be lost and HOG descriptor is sensitive to the leaf petiole orientation while the petiole’s shape actually carries the species characteristics. Therefore, to overcome these issues, a pre-processing step can normalize petiole orientation of all leaf images in a dataset making them practicable to HOG. This idea had been proposed by two studies, (Cope et al., 2012) and (Xiao et al., 2010), and it was proven that HOG achieved a better performance when the leaf petiole was cut off before analysis (Xiao et al., 2010).

On the other hand, Hu invariant moments obtained the worst classification accuracy. Even though Hu moments were computationally simple, it was highly sensitive to noise. Seven Hu invariant moments can describe shape characteristics well, but seven descriptors were not enough for feature extraction because the information carried by their own were very restricted when the image database is large. They usually need to be combined with other conventional descriptors in order to better describe the actual shape properties of the object.

Zernike moments obtained relatively good results of classification accuracy and it can be a feasible alternative for classifying structural complex images. Zernike moments provided exceptional invariance features over other moment based solution like the Hu invariant moments. However, the limitation of Zernike moments was the costly computation that made it inapt for some problems.

From the results in Table 4, the classification accuracy increased by combining more descriptors. When only MSD descriptor were used, false classification rate increased for similar shaped leaves of some species. The leaf samples of species 24 (*Mussaenda erythrophylla*) and species 25 (*Mussaenda philippica*) (refer to Fig. 4) were often misclassified when MSD was used as input descriptors only. This is due to the shape of the leaves being similar to each other, since both of them belong to the same genus but different species. Although these leaves have similar shapes, the leaf petiole for both species were obviously different.

The classification accuracy increased using a hybridisation of MSD and HOG descriptor and this helped to decrease the misclassification of these species, as HOG descriptor was sensitive to the petiole orientation. The hybrid of two and three descriptors achieved almost comparable results of the classification accuracy. Subsequently, the hybridisation of all descriptors performed the best results compared to single, two or three descriptors. The MSD and HOG descriptors were the major contributors in the classification of tropical shrub species. Hu invariant moments and Zernike moments descriptors, on the other hand, helped to improve the classification accuracy in tropical shrub species in terms of invariant to translation, rotation, and scale.

Feature selection does not necessarily mean an increase in accuracy. In fact, in all cases, reducing the number of descriptors too dramatically (more than 60.15% reduction), will result in a decrease in the accuracy. However, based on the results obtained in Table 5, it has been shown that the reduction in descriptors, the feature selection methods of Relief, CFS, and PCC achieved comparable results compared to using all 133 descriptors, The Relief method achieved the best result of 98.13% with 53 descriptors (60% reduction) and reduced computational time by 1,033.23 min, if compared to model with all descriptors. This proved that feature selection methods are able to select the optimal descriptors that are correlated to each other in the classification of tropical shrub species.

Finally, the performance of our proposed method compared to other leaf classification studies is shown in Table 8. In the study performed by Pham et al. (2013), they compared the HOG and Hu invariant moments, and the results showed that the HOG descriptor was more robust than the Hu invariant moments descriptor. The accuracy of the HOG and Hu invariant moments descriptor achieved in this study were 84.70% and 25.31% respectively. In the study presented by Salve et al. (2016), the implementation of the HOG and Zernike moments descriptor were proposed. This study used the subset Visleaf dataset which contained 50 plant species and 10 samples for each of species, which is a total of 500 images. By using the Zernike moments as descriptor, the accuracy achieved 84.66% whereas HOG descriptor achieved 92.67%, and this indicated that Zernike moments had lower accuracy compared to HOG. Wu et al. (2007) used geometrical descriptors and morphological descriptors in the vein structure. The algorithm was quite simple and provided a good result of 90.31% of accuracy but it required human intervention for the physiological length width. Moving on, Du, Wang & Zhang (2007) used a combination of morphological and Hu invariant moments to recognize 20 species of plant and achieved 91% accuracy. Hossain & Amin (2010) used only MSD as part of the descriptors set and obtained around 93% of accuracy.

**Table 8:** Comparison studies.

Reference	Descriptor	Leaf dataset	Accuracy
Pham et al. (2013)	HOG	Flavia	84.70%
	Hu invariant moments		25.31%
Salve et al. (2016)	Zernike moments	Visleaf	84.66%
	HOG		92.67%
Wu et al. (2007)	MSD	Flavia	90.31%
Du, Wang & Zhang (2007)	MSD, Hu invariant moments	Own dataset	91.00%
Hossain & Amin (2010)	MSD	Flavia	91.41%
Kadir et al. (2011)	MSD, Texture, Color	Flavia	93.75%
Kulkarni et al. (2013)	MSD, Zernike moments, Vein, Color, Texture	Flavia	93.82%
Priya, Balasaravanan & Thanamani (2012)	MSD, Vein	Flavia	94.20%
Current study	MSD, HOG, Hu invariant moments, Zernike moments	MyDAUN	98.23%
		Flavia	95.25%
		Swedish Leaf	99.89%

Kulkarni et al. (2013) and Kadir et al. (2011) used MSD, Zernike moments, colour moments and texture as part of the descriptors set. Colour and texture were not expected to be as descriptive as shape for leaf analysis since most leaves are in the same shade of green that also change greatly under different illumination (Yanikoglu, Aptoula & Tirkaz, 2014). In addition, there are low inter-class variability in terms of colour and high intra class variability. Even the colours of leaves belonging to the same species or even plant can present a wide range of colours depending on the season. For instance, most dried leaves change colour, therefore it is not commonly used as an important descriptor for leaf analysis. The vein structure of a leaf is unique to a species. It has higher contrast compared to the rest of the leaf blade and is often visible (Priya, Balasaravanan & Thanamani, 2012). The combination of MSD with vein gave a recognition accuracy of 94.20%. The previous studies showed that most of the algorithms used PNN, k-NN, SVM, or RF as a classifier. Among these approaches, ANN has the fastest speed and best accuracy for classification. Suk, Flusser & Novotný (2013) showed that the ANN classifier ran faster than the k-NN and MCC hypersphere classifier with a higher accuracy. Therefore, ANN was adopted as a classifier in this study.

## Conclusion

In this study, a classification of a tropical shrub species using a hybrid of shape approach and machine learning was developed. The results showed that the hybrid of four descriptors of MSD, HOG, Hu invariant moments, and Zernike moments stands out to be comparably better than single, two and three descriptors. The classification of tropical shrub species was conducted using six different classifiers, which are, ANN, RF, SVM, k-NN, LDA and DAG MLSTSVM. Relief, CFS and PCC feature selection techniques were tested to determine their effectiveness for reducing the number of descriptors in the myDAUN dataset. In addition, validation was done using the Flavia and Swedish Leaf datasets to consolidate the obtained results, the results showed that the hybrid of all 133 descriptors of ANN outperformed the other classifier with an average classification accuracy of 98.23% for the myDAUN dataset, 95.25% for the Flavia dataset and 99.89% for the Swedish Leaf dataset. Furthermore, the Relief feature selection method achieved the highest classification accuracy of 98.13% with 53 descriptors and 54.35% reduction of computational time. This study found that Relief is a highly effective method for feature selection, which had both reduced the number of dimensions for the dataset, gave comparable results of accuracy with reduction in the computation time. The results also showed that the optimal descriptors are MSD and HOG whereas Hu invariant moments and Zernike moments helped to improve the classification accuracy in terms of invariant to translation, rotation and scale.

##  Supplemental Information

10.7717/peerj.3792/supp-1 Supplemental Information 1 myDAUN dataset Thumbnail view of myDAUN dataset.

## Additional Information and Declarations

### Data Availability

The following information was supplied regarding data availability: Chang, Siow-Wee; Murat, Mirae (2017): myDAUN dataset. figshare.
https://doi.org/10.6084/m9.figshare.5259643.v1.

## References

[ref-1] Aakif A, Khan MF (2015). Automatic classification of plants based on their leaves. Biosystems Engineering.

[ref-2] Ahmed N, Khan UG, Asif S (2016). An automatic leaf based plant identification.

[ref-3] Alpaydin E (2014). Introduction to machine learning.

[ref-4] Breiman L (2001). Random forests. Machine Learning.

[ref-5] Chaki J, Parekh R, Bhattacharya S (2015). Plant leaf recognition using texture and shape features with neural classifiers. Pattern Recognition Letters.

[ref-6] Cope JS, Corney D, Clark JY, Remagnino P, Wilkin P (2012). A review of plant species identification using digital morphometrics. Expert Systems with Applications.

[ref-7] Corlett RT (2016). Plant diversity in a changing world: status, trends, and conservation needs. Plant Diversity.

[ref-8] Cortes C, Vapnik V (1995). Support-vector networks. Machine Learning.

[ref-9] Dalal N, Triggs B (2005). Histograms of oriented gradients for human detection.

[ref-10] Du J-X, Huang D-S, Wang X-F, Gu X (2006). Computer-aided plant species identification (capsi) based on leaf shape matching technique. Transactions of the Institute of Measurement and Control.

[ref-11] Du J-X, Wang X-F, Zhang G-J (2007). Leaf shape based plant species recognition. Applied Mathematics and Computation.

[ref-12] Fu H, Chi Z (2006). Combined thresholding and neural network approach for vein pattern extraction from leaf images.

[ref-13] Geertsema M, Highland L, Vaugeouis L, Sassa K, Canuti P (2009). Environmental impact of landslides. Landslides—disaster risk reduction.

[ref-14] Hall MA (1999). Correlation-based feature selection for machine learning.

[ref-15] Hore AV, Kehoe JG, McMullan R, Penton MR (1997). Development of the built environment. construction 2: environment science materials technology.

[ref-16] Hossain J, Amin MA (2010). Leaf shape identification based plant biometrics.

[ref-17] Hu M-K (1962). Visual pattern recognition by moment invariants. Information Theory, IRE Transactions.

[ref-18] Hwang SK, Kim WY (2006). A novel approach to the fast computation of Zernike moments. Pattern Recognition.

[ref-19] Kadir A, Nugroho LE, Susanto A, Santosa PI (2011). A comparative experiment of several shape methods in recognizing plants. International Journal of Computer Science & Information Technology.

[ref-20] Kadir A, Nugroho LE, Susanto A, Santosa PI (2012). Experiments of Zernike moments for leaf identification. Journal of Theoretical and Applied Information Technology.

[ref-21] Kellogg EA (2015). Flower structure. Flowering plants monocots: poaceae.

[ref-22] Kulkarni A, Rai H, Jahagirdar K, Kadkol R (2013). A leaf recognition system for classifying plants using RBPNN and pseudo Zernike moments. International Journal of Latest Trends in Engineering and Technology.

[ref-23] Kumar N, Belhumeur PN, Biswas A, Jacobs DW, Kress WJ, Lopez IC, Soares JVB, Fitzgibbon A, Lazebnik S, Perona P, Sato Y, Schmid C (2012). Leafsnap: a computer vision system for automatic plant species identification.

[ref-24] Mata-Montero E, Carranza-Rojas J, Mata FJ, Pont A (2016). Automated plant species identification: challenges and opportunities.

[ref-25] Menges ES, Fiedler PL, Kareiva PM (1998). Evaluating extinction risks in plant populations. Conservation biology: for the coming decade.

[ref-26] Oncevay-Marcos A, Juarez-Chambi R, Khlebnikov-Núñez S, Beltrán-Castañón C, Azzopardi G, Petkov N (2015). Leaf-based plant identification through morphological characterization in digital images.

[ref-27] Pham NH, Le TL, Grard P, Nguyen VN (2013). Computer aided plant identification system.

[ref-28] Pornpanomchai C, Rimdusit S, Tanasap P, Chaiyod C (2011). Thai herb leaf image recognition system (THLIRS). Kasetsart Journal (Natural Science).

[ref-29] Priya CA, Balasaravanan T, Thanamani AS (2012). An efficient leaf recognition algorithm for plant classification using support vector machine.

[ref-30] Rademaker CA (2000). The classification of plants in the United States Patent Classification system. World Patent Information.

[ref-31] Salve P, Sardesai M, Manza R, Yannawar P, Satapathy SC, Raju KS, Mandal JK, Bhateja V (2016). Identification of the plants based on leaf shape descriptors.

[ref-32] Shanwen Z, YouQian F (2010). Plant leaf classification using plant leaves based on rough set.

[ref-33] Sharma S, Gupta C (2015). Recognition of plant species based on leaf images using multilayer feed forward neural network. International Journal of Innovative Research in Advanced Engineering.

[ref-34] Singh S, Haddon J, Markou M (2001). Nearest-neighbour classifiers in natural scene analysis. Pattern Recognition.

[ref-35] Suk T, Flusser J, Novotný P, Wilson R, Hancock E, Bors A, Smith W (2013). Comparison of leaf recognition by moments and fourier descriptors.

[ref-36] Teague MR (1980). Image analysis via the general theory of moments*. Journal of the Optical Society of America.

[ref-37] Tilman D, Cassman KG, Matson PA, Naylor R, Polasky S (2002). Agricultural sustainability and intensive production practices. Nature.

[ref-38] Tomar D, Agarwal S (2016). Leaf recognition for plant classification using direct acyclic graph based multi-class least squares twin support vector machine. International Journal of Image and Graphics.

[ref-39] University of Malaya (1999). Rimba Ilmu Botanic Garden. http://rimba.um.edu.my/.

[ref-40] Viscosi V, Cardini A (2011). Leaf morphology, taxonomy and geometric morphometrics: a simplified protocol for beginners. PLOS ONE.

[ref-41] Wang X-F, Huang D-S, Du J-X, Xu H, Heutte L (2008). Classification of plant leaf images with complicated background. Applied Mathematics and Computation.

[ref-42] Wiens JJ (2016). Climate-related local extinctions are already widespread among plant and animal species. PLOS Biology.

[ref-43] Wu SG, Bao FS, Xu EY, Wang Y-X, Chang Y-F, Xiang Q-L (2007). A leaf recognition algorithm for plant classification using probabilistic neural network.

[ref-44] Xiao X-Y, Hu R, Zhang S-W, Wang X-F (2010). HOG-based approach for leaf classification.

[ref-45] Yanikoglu B, Aptoula E, Tirkaz C (2014). Automatic plant identification from photographs. Machine Vision and Applications.

