# Supplementary material for: Automated classification of tropical shrub species: a hybrid of leaf shape and machine learning approach
Source: PeerJ. 2017 Sep 12;5:e3792. doi: 10.7717/peerj.3792 (PMC5600178; doi:10.7717/peerj.3792)

Species 1 – ACALYPA SIAMENSIS

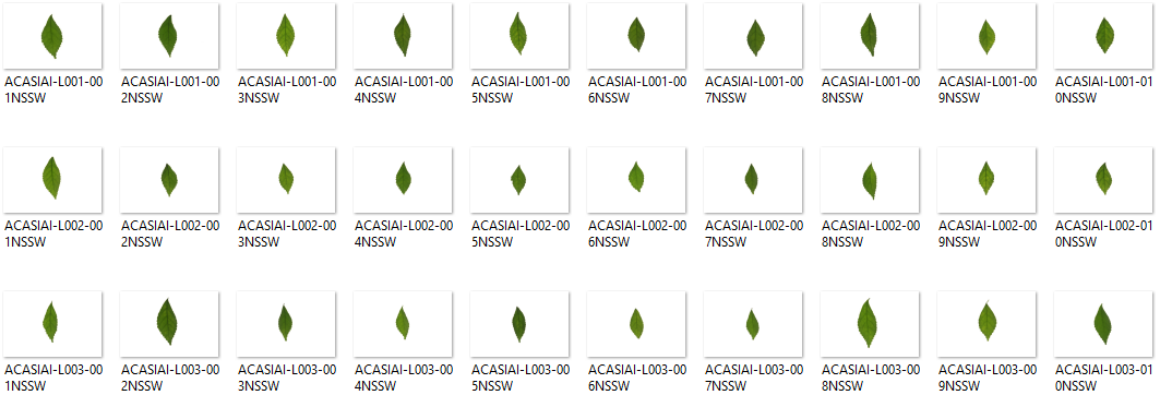

Species 2 – ACALYPHA WILKESIANA

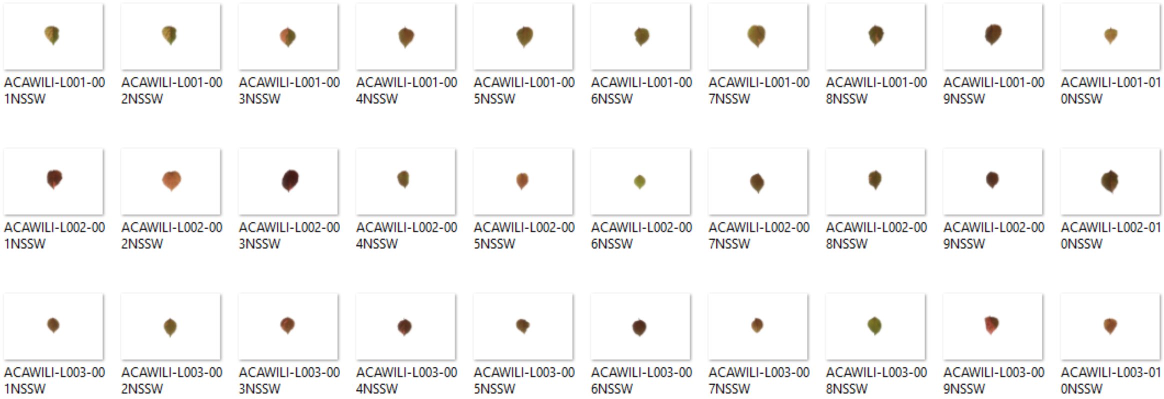

Species 3 – ALLAMANDA CATHARTICA

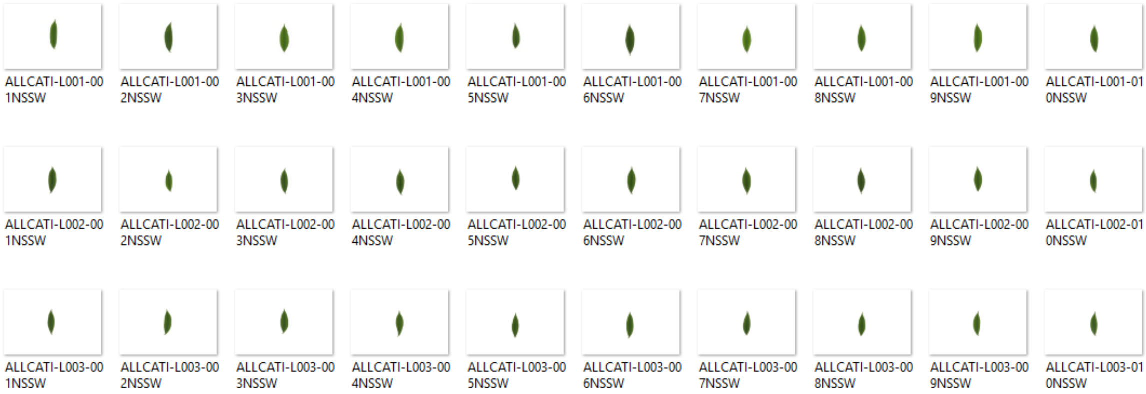

Species 4 – BOUGAINVILLEA SPECTABILIS

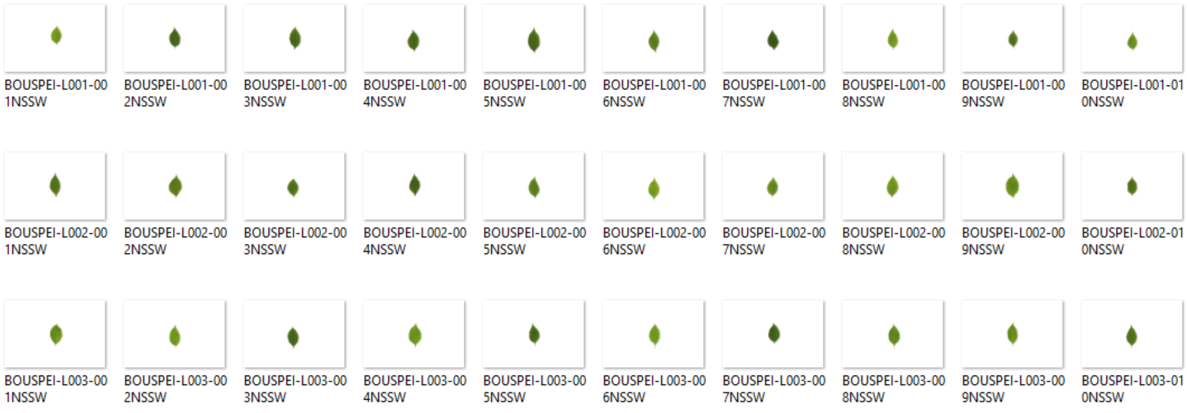

Species 5 – BRUNTELSIA CALYCINA

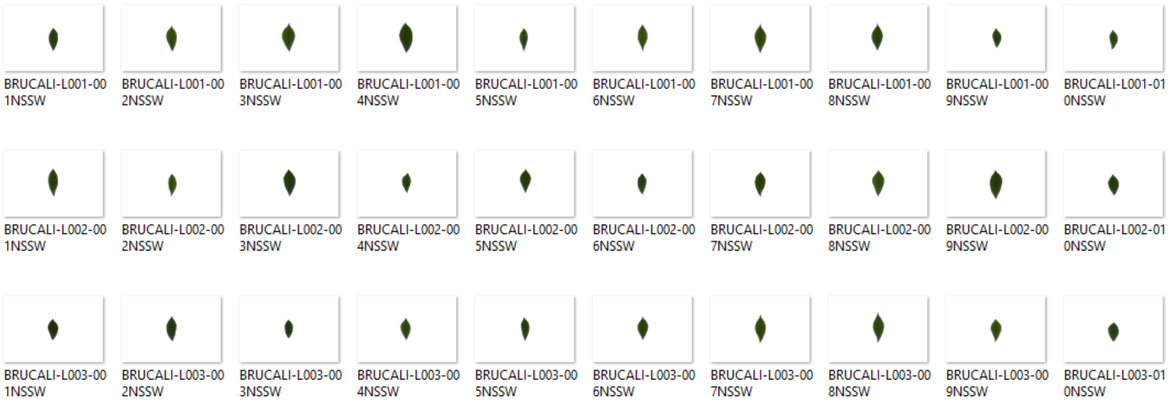

Species 6 – CLINACANTHUS NUTANS

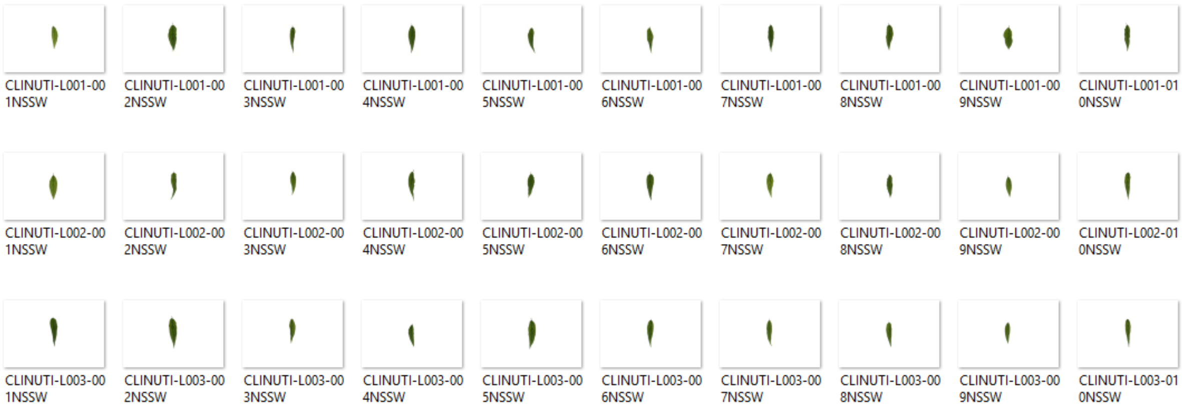

Species 7 –

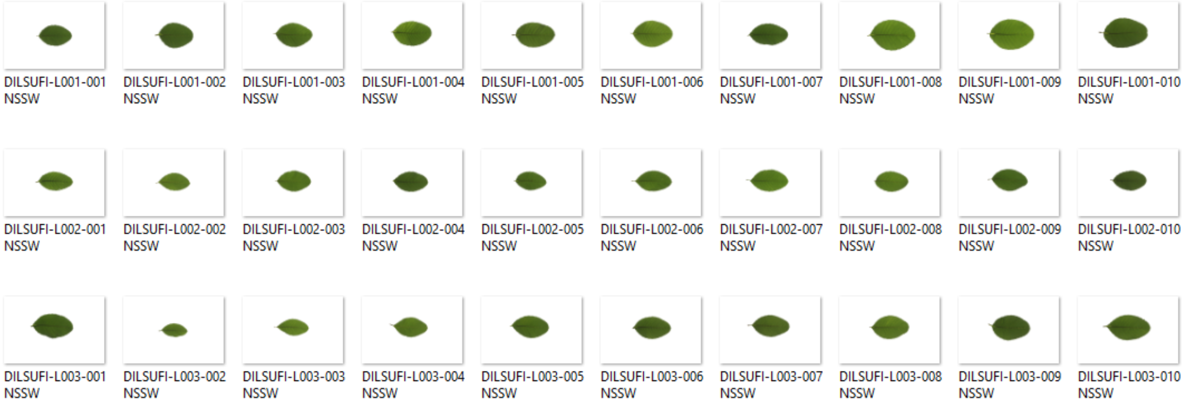

Species 8 – DRACAENA REFLEXA

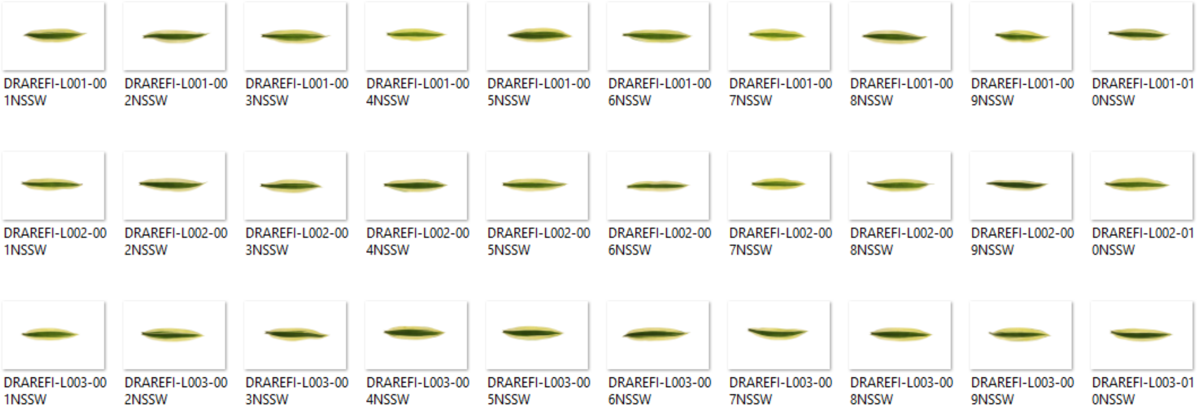

Species 9 – DRACAENA SURCULOSA

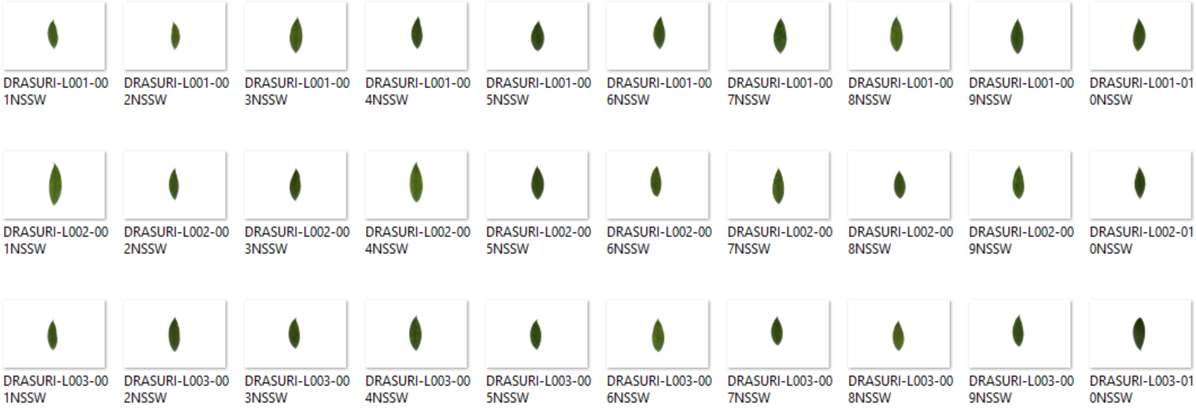

Species 10 – DURANTA ERECTA

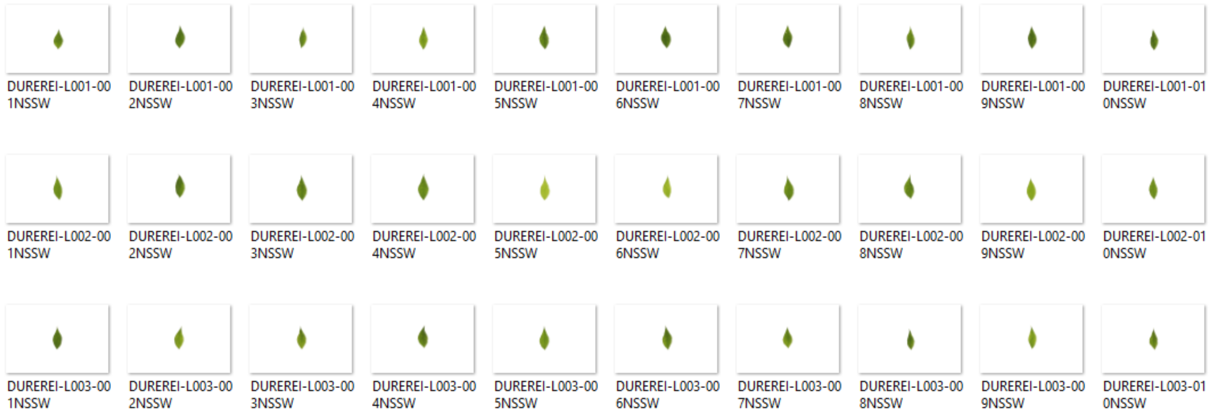

Species 11 – EXCOECARIA COCHINCHINENSIS

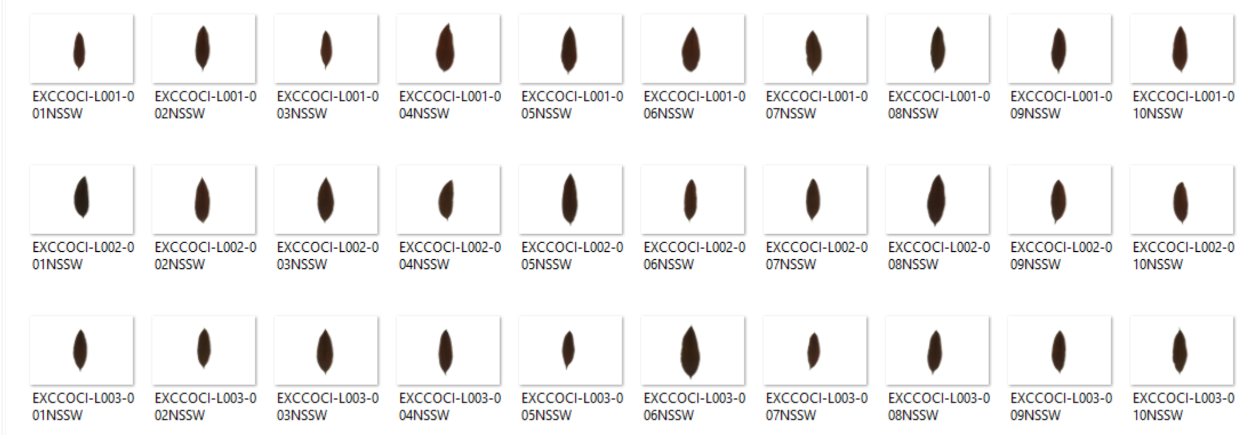

Species 12 – GRAPTOPHYLLUM PICTUM

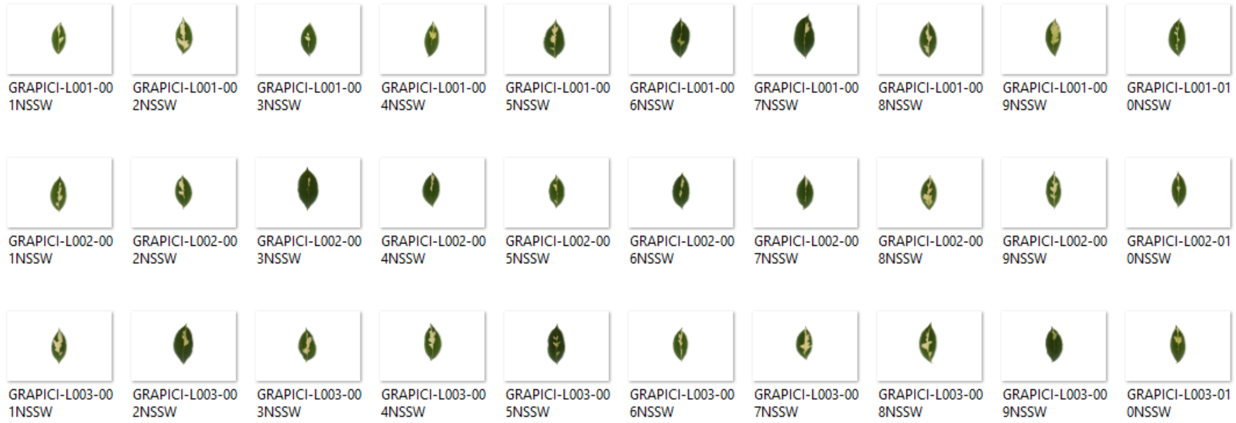

Species 13 – HIBICUS ROSA-SINENSIS

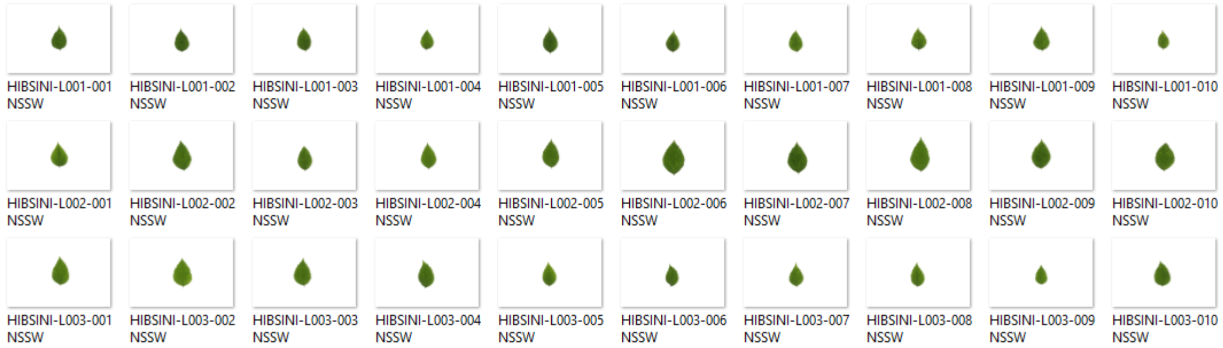

Species 14 – IXORA JAVANICA

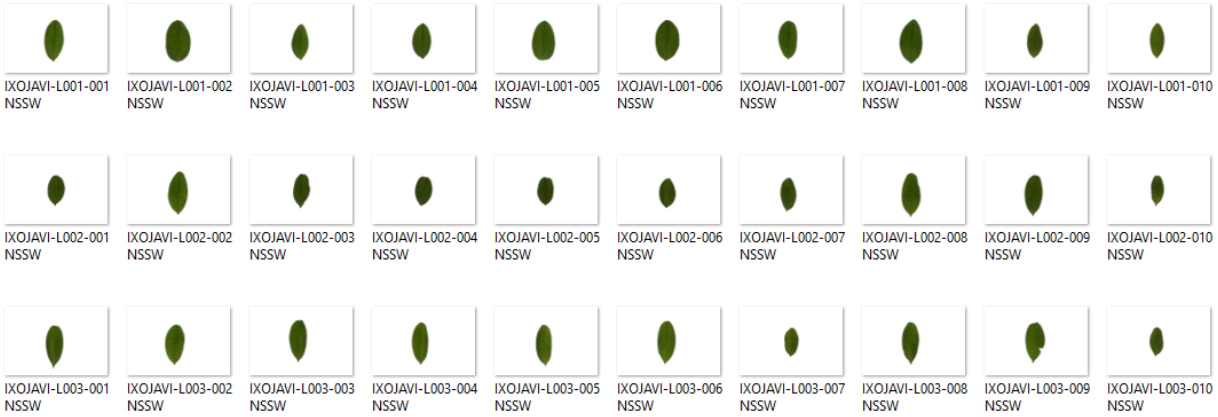

Species 15 – LAGERSTROEMIA INDICA

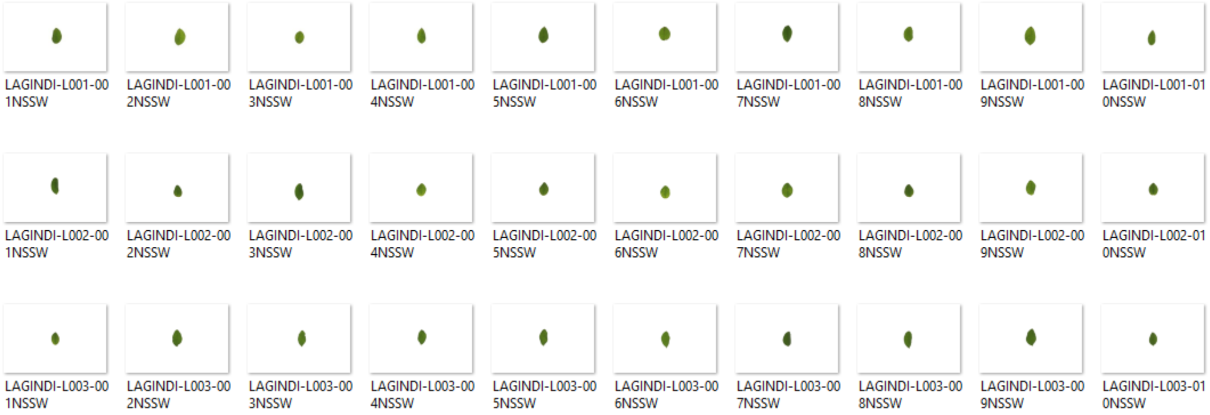

Species 16 – LANTANA CAMARA

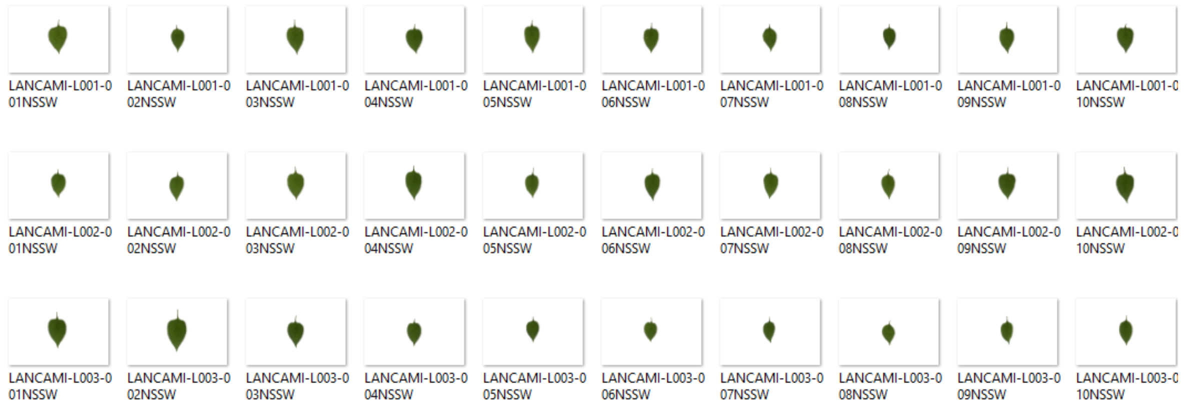

Species 17 – LAWSONIA INERMIS

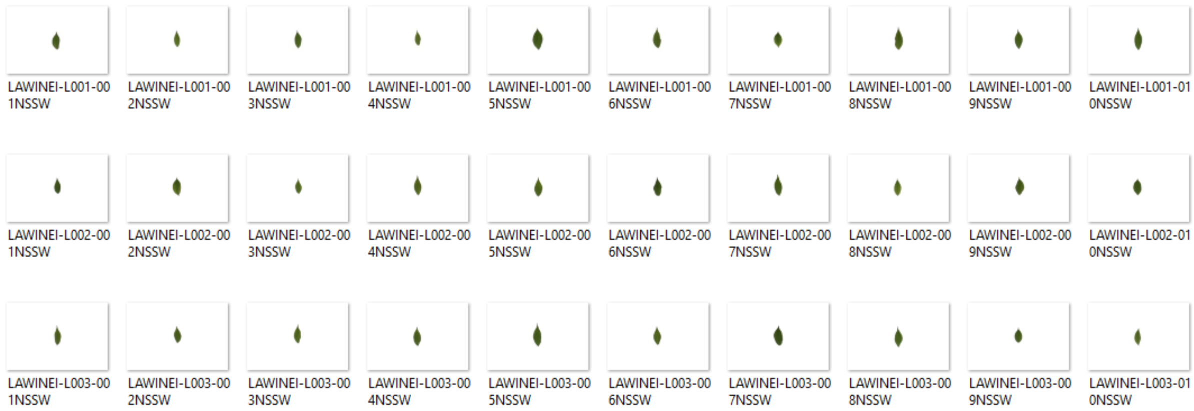

Species 18 – LOROPETALUM CHINENSE

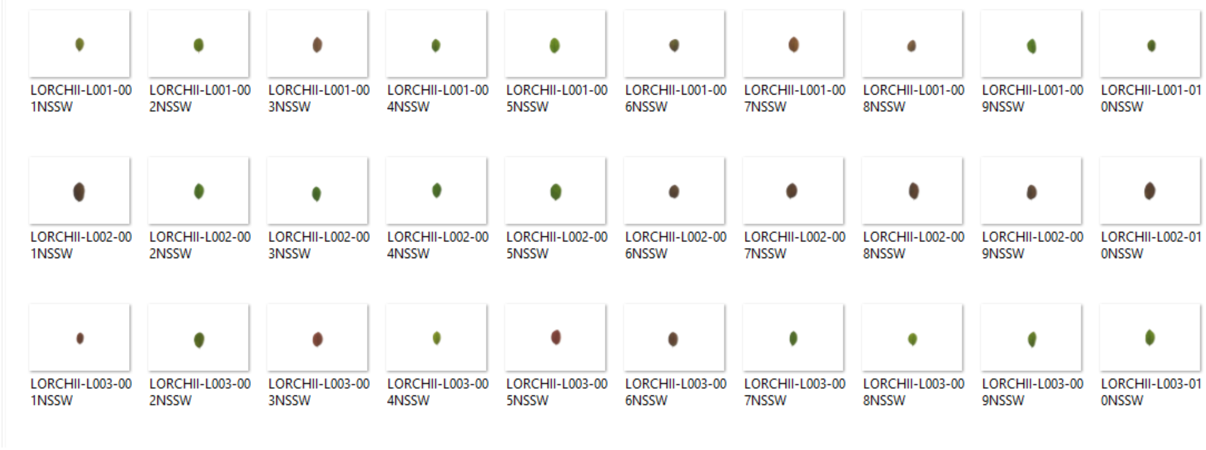

Species 19 – MAGNOLIA FIGO

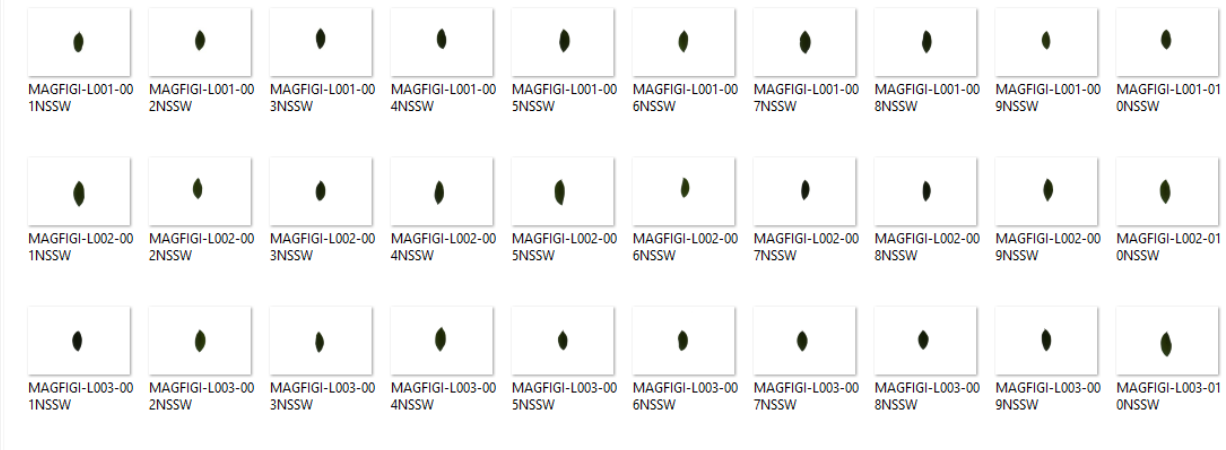

Species 20 –

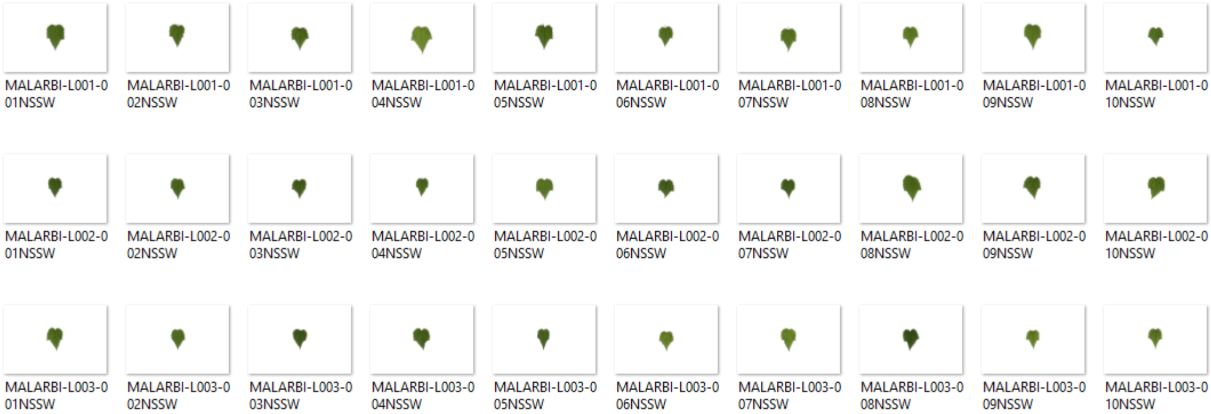

Species 21 – MANIHOT ESCULENTA

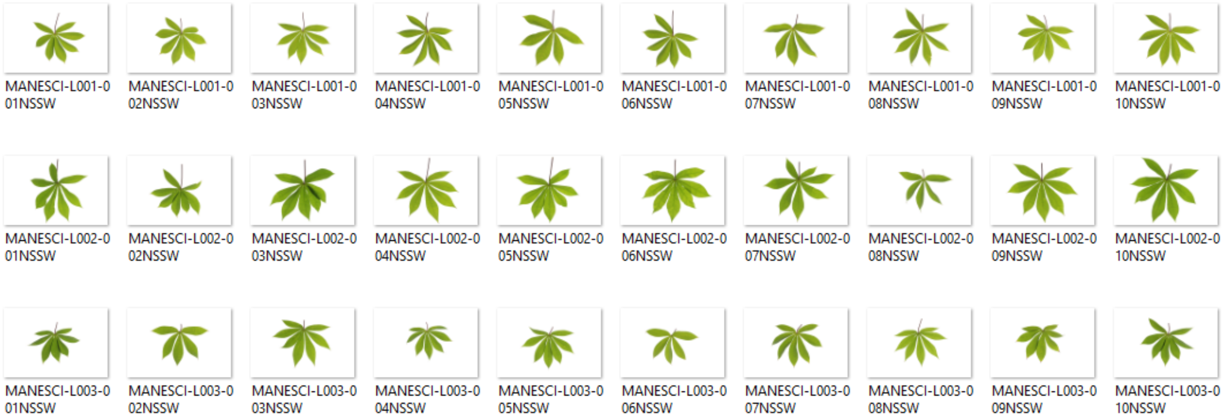

Species 22 – MELASTROMA MALABATHRICUM

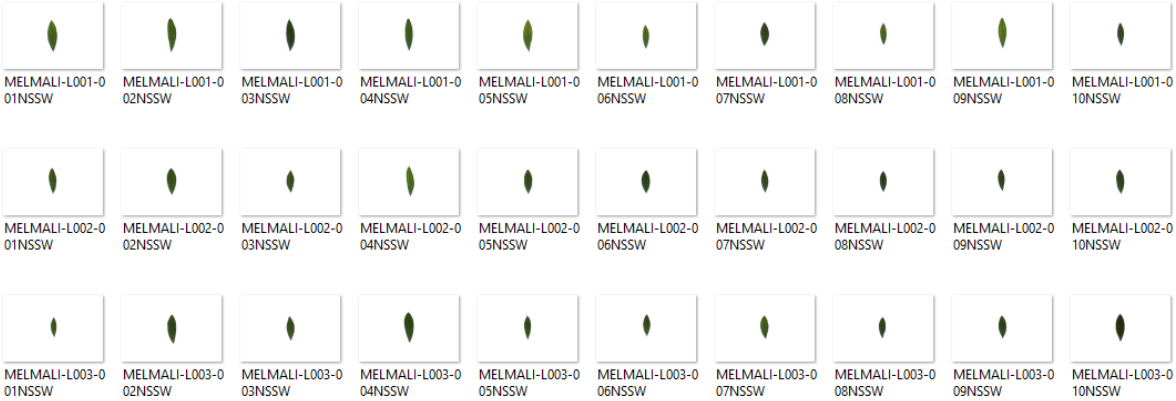

Species 23 – MURRAYA PANICULATA

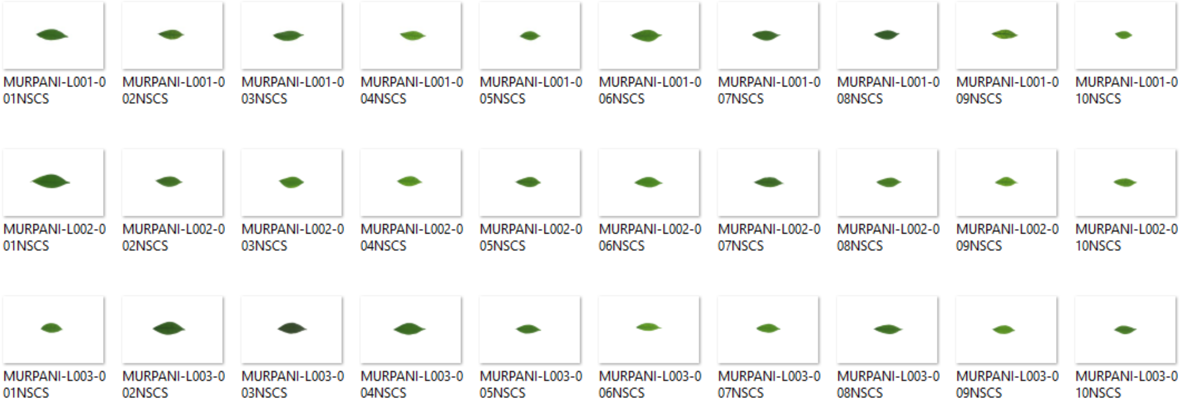

Species 24 – MUSSAENDA ERYTHROPHYLLA

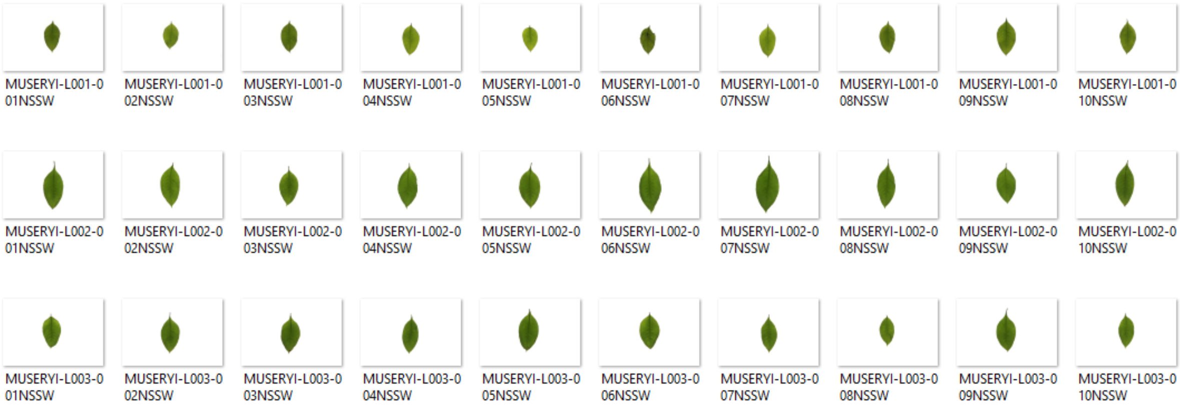

Species 25 – MUSSAENDA PHILLIPICA

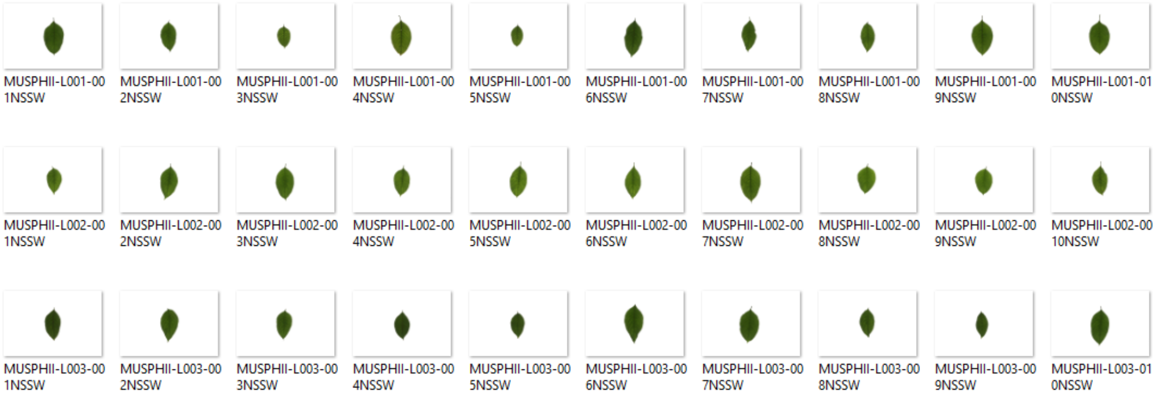

Species 26 – PHYLLANTUS MYRTIFOLIUS

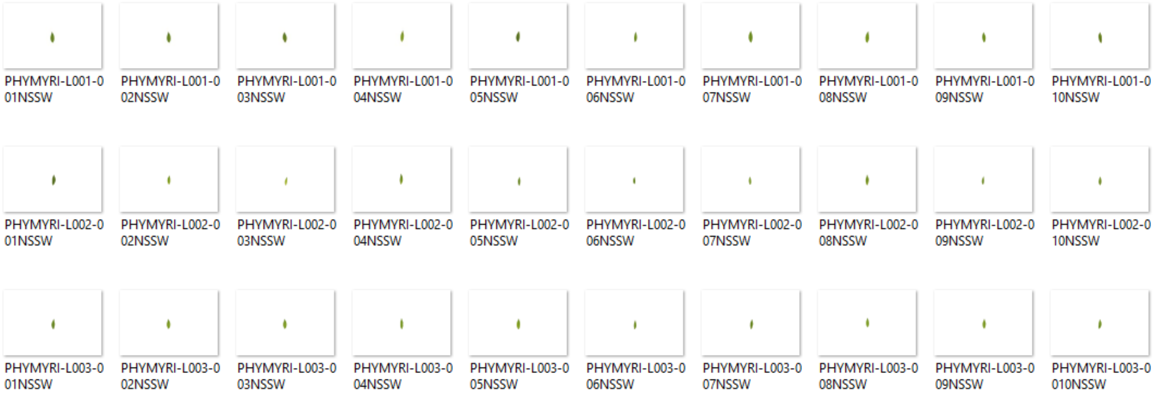

Species 27 – POLYSCIAS BALFOURIANA

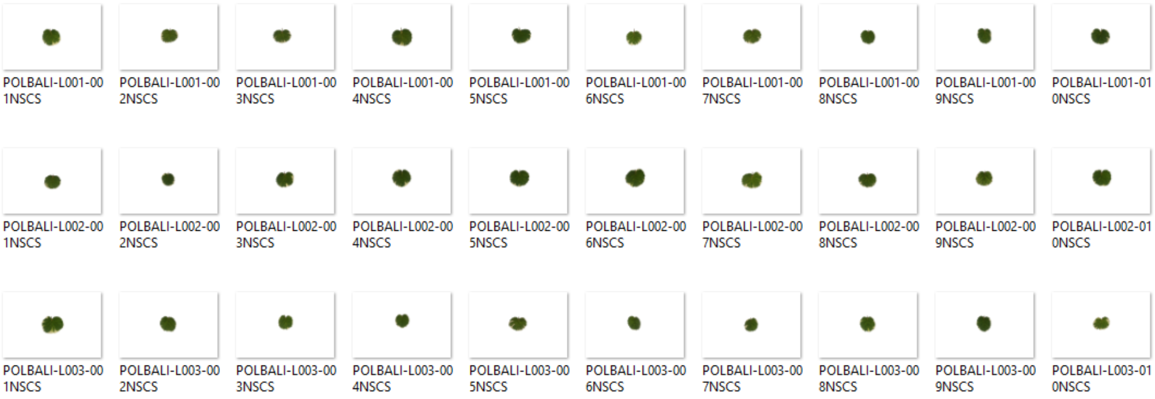

Species 28 – SAUROPUS ANDROGYNUS

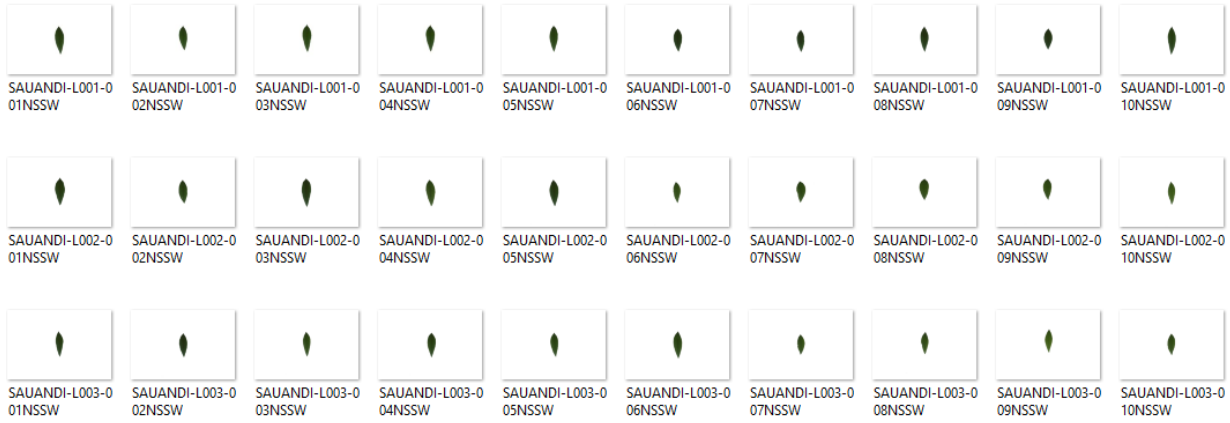

Species 29 – STROBILANTHES CRISPA

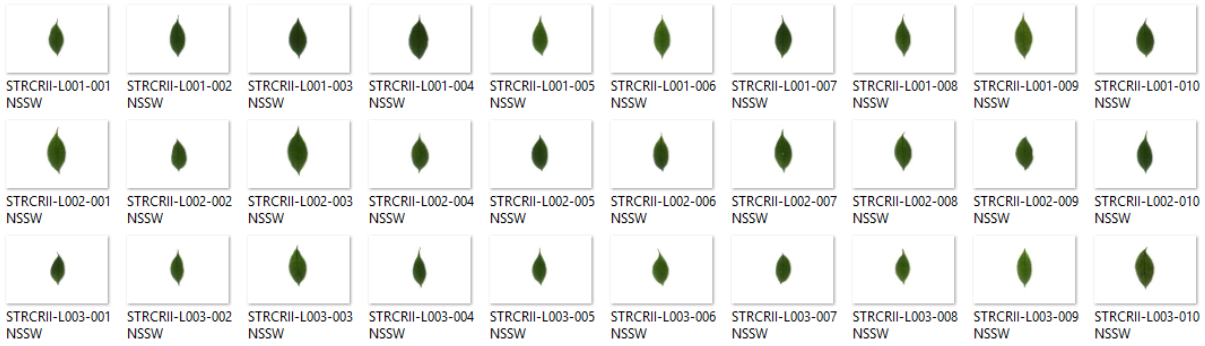

Species 30 – TABERNAEMONTANA DIVARICATE

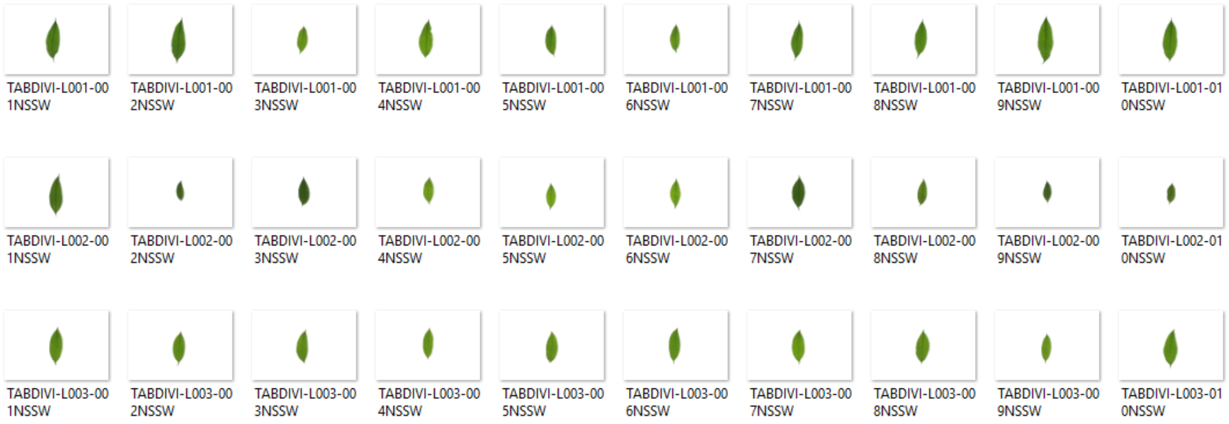

Species 31 – TIBOUCHINA URVILLEANA

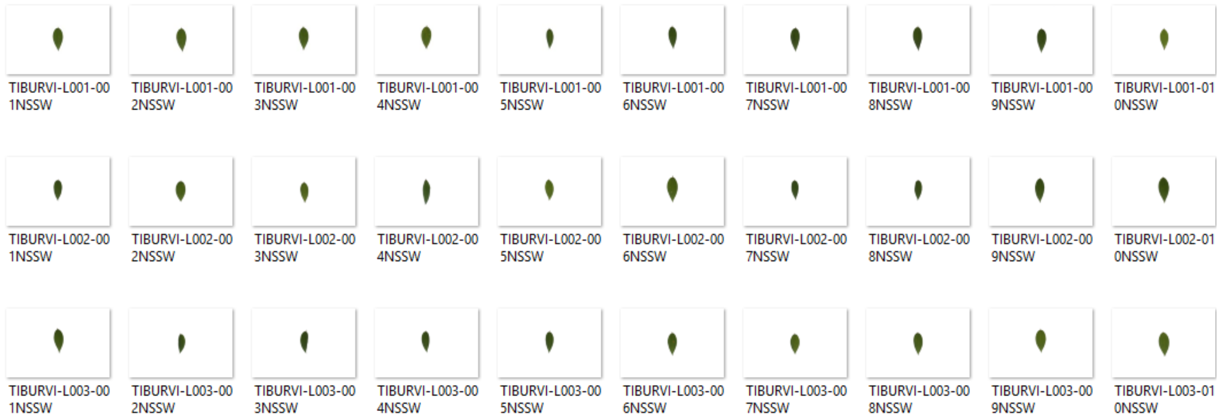

Species 32 – CITRUS MICROCARPA

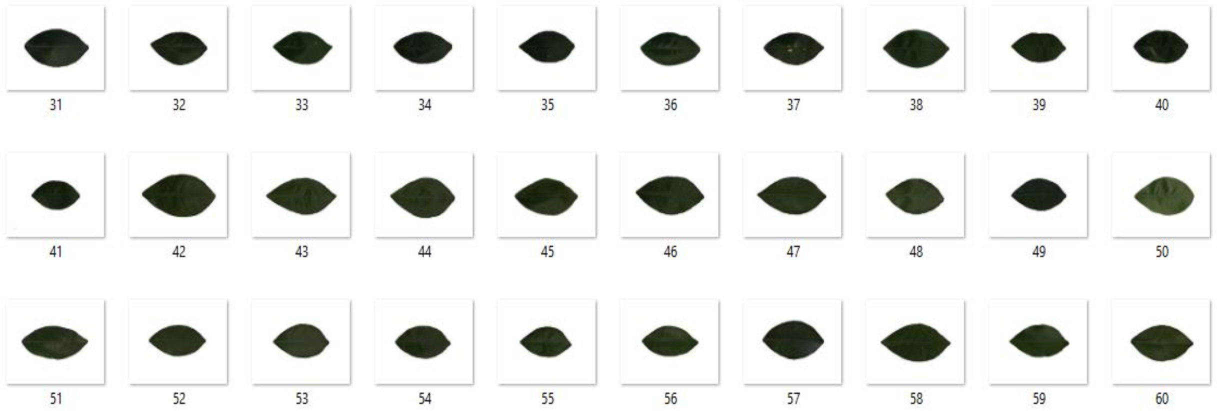

Species 33 – MENTHA PIPERITA

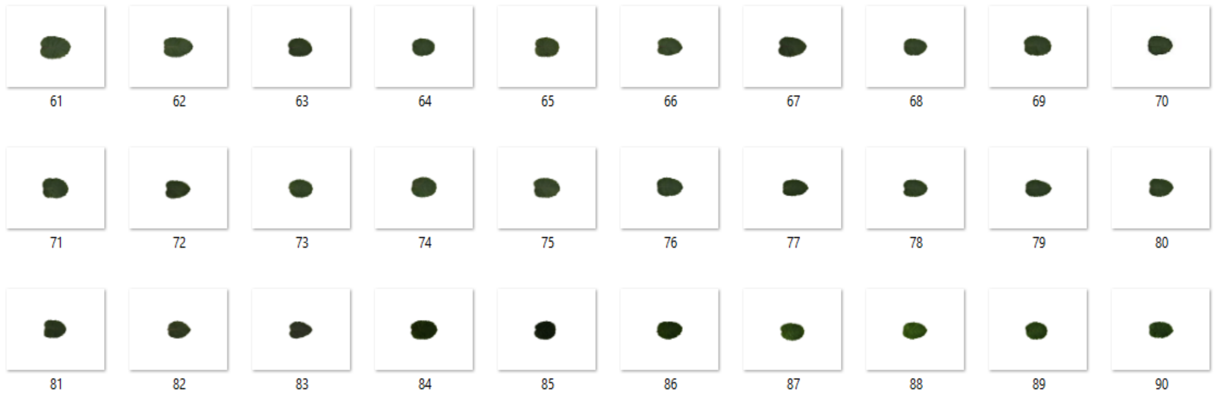

Species 34 – ANDROGRAPHIS PANICULATA

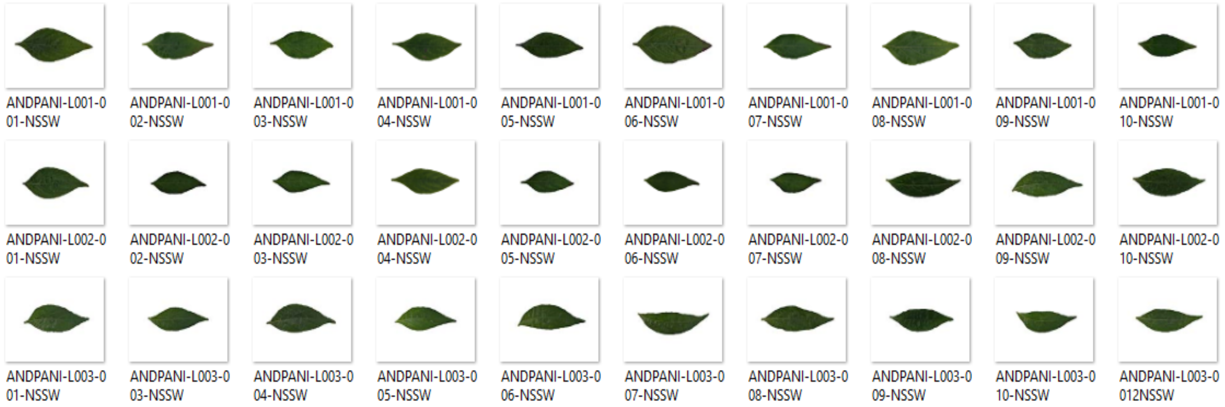

Species 35 – RHODOMYRTUS TOMENTOSA

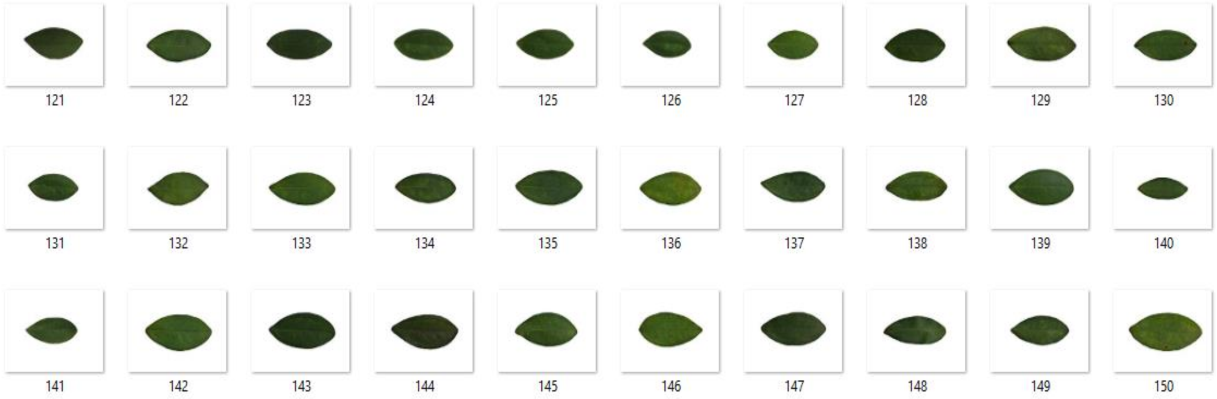

Species 36 – ORTHOSIPHON ARISTATUS

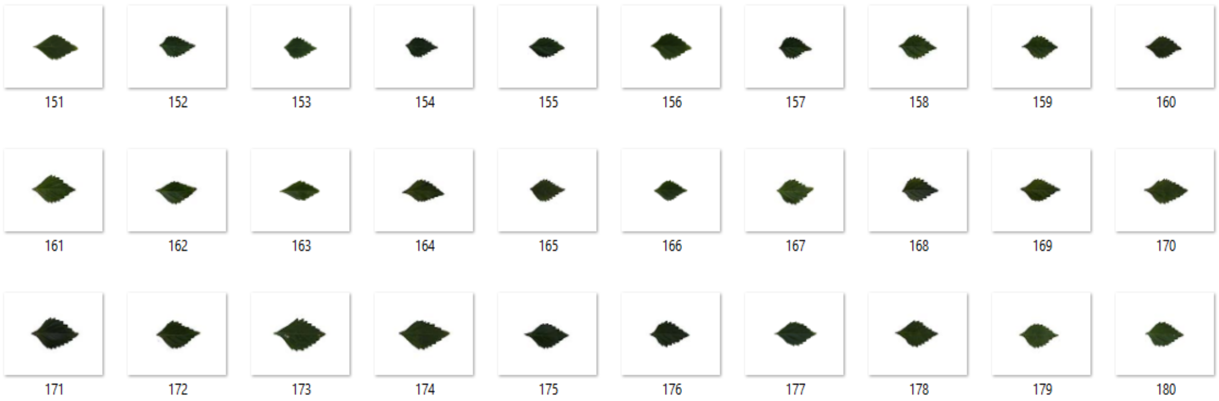

Species 37 – CENTRATHERUM PUNCTATUM

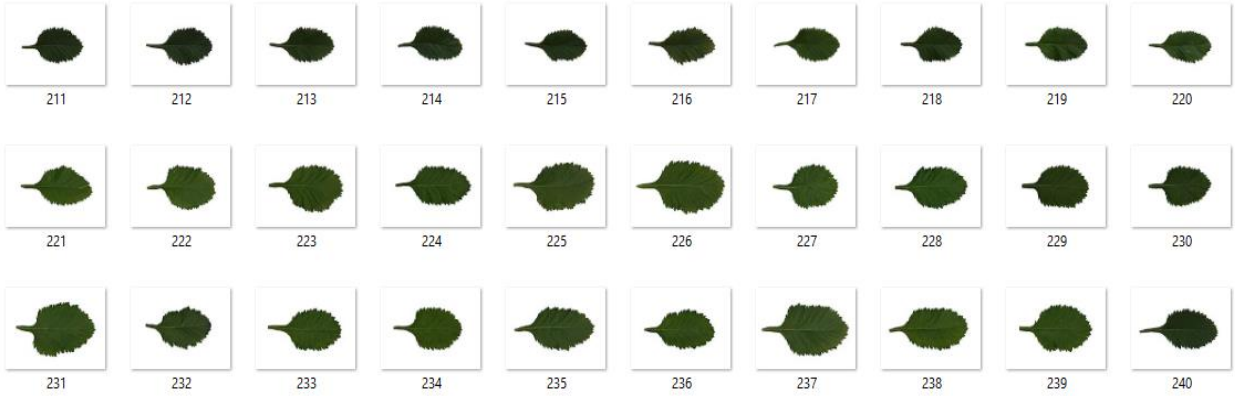

Species 38 – POLYGONUM MINUS

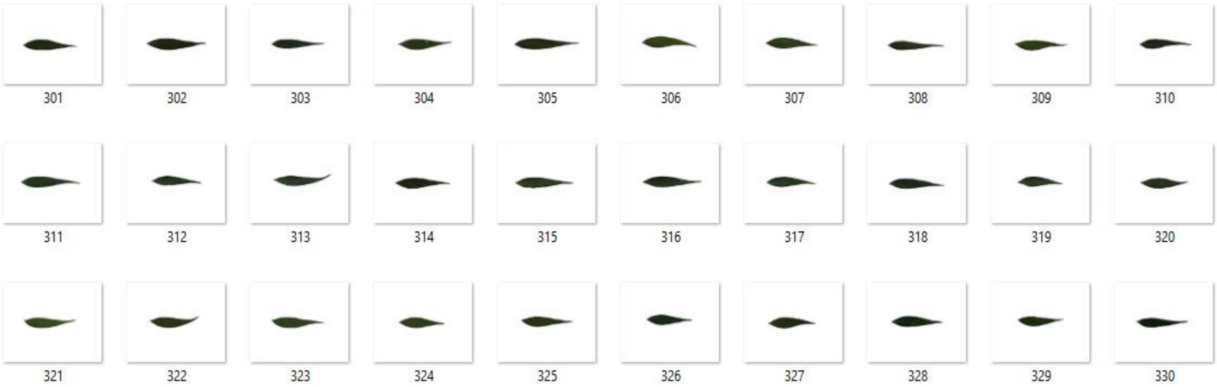

Species 39 – TABERNAEMONTANA CORONARIA

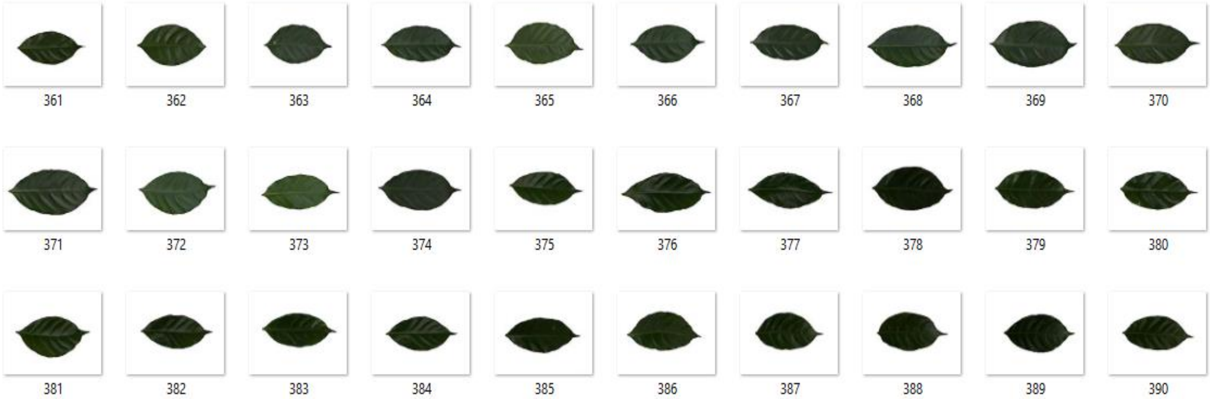

Species 40 – JUSTICIA GENDARUSA

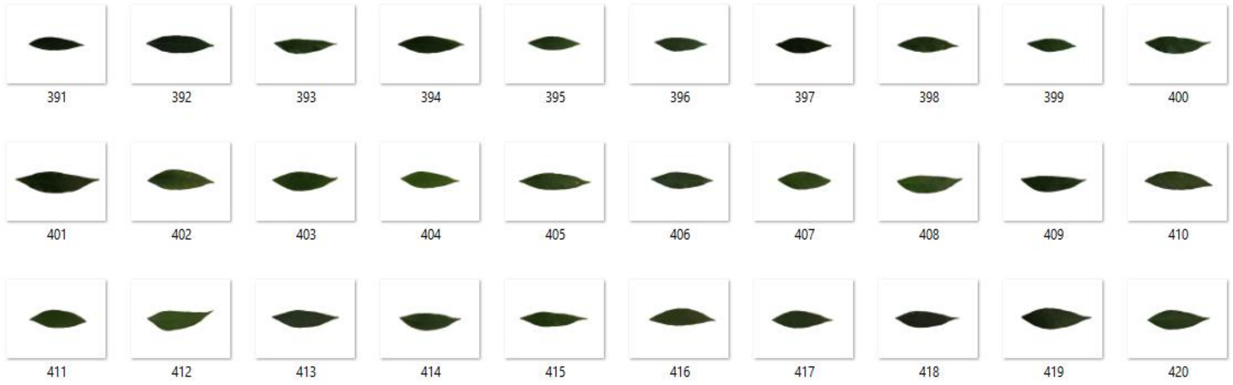

Species 41– TETRACERA SCANDENS

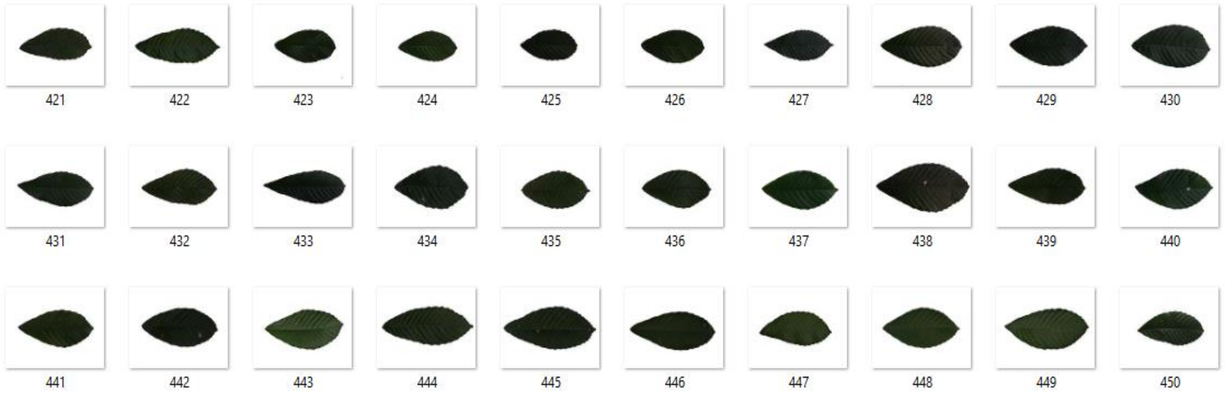

Species 42 – PIPER SARMENTOSUM

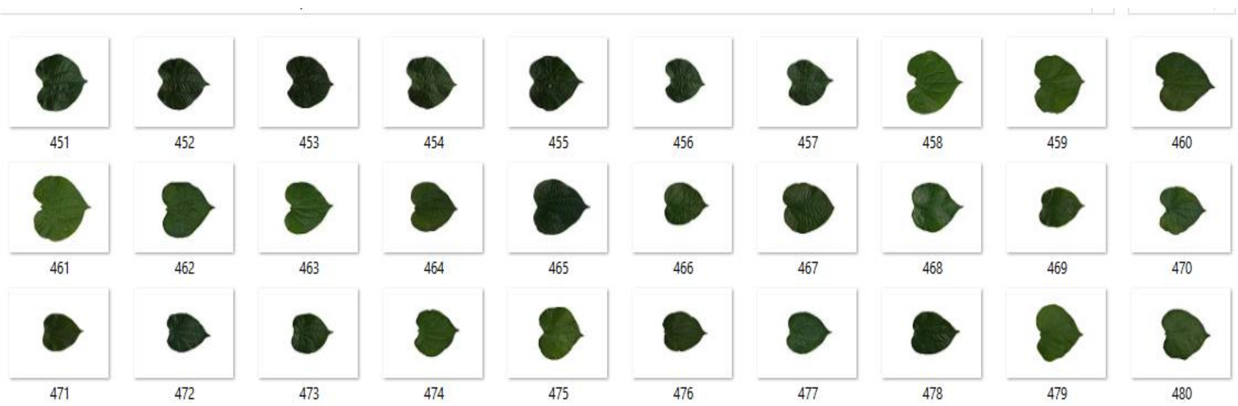

Species 43 – RAUVOLFIA SERPENTINA

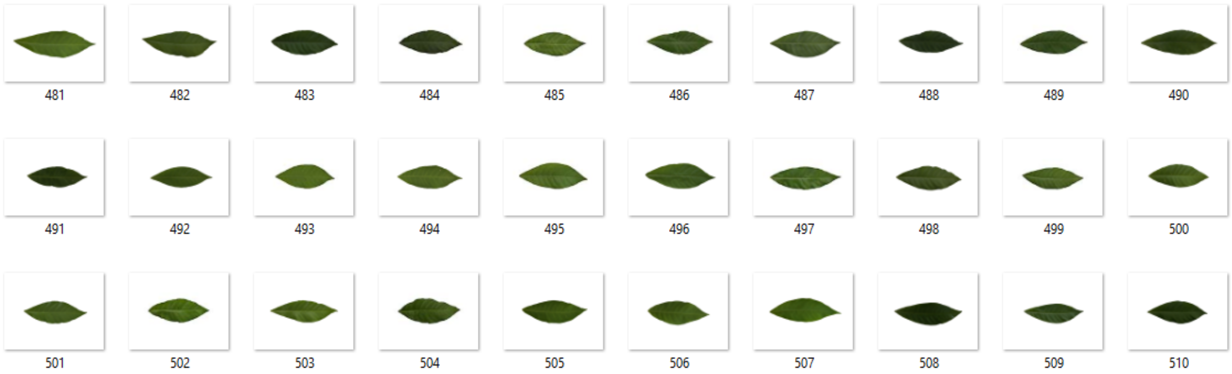

Species 44 – FLEMINGIA STROBILIFERA

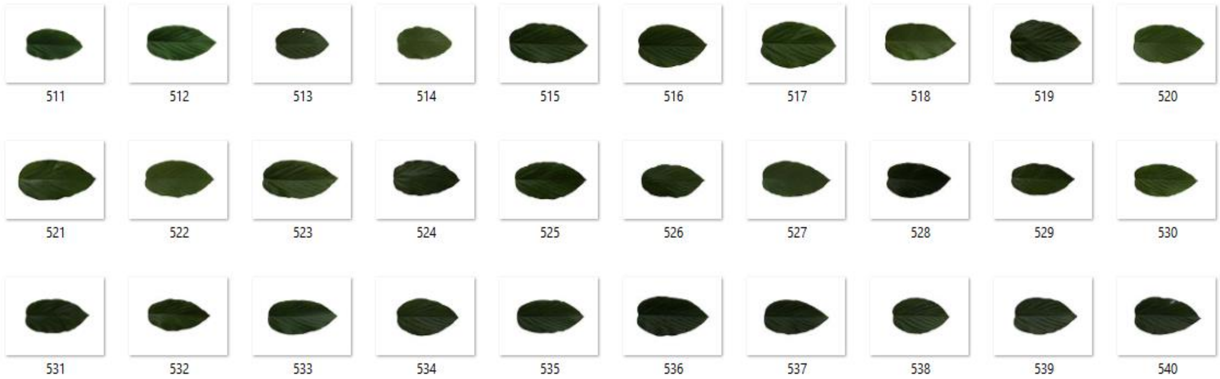

Species 45 – CANANGA ODORATA

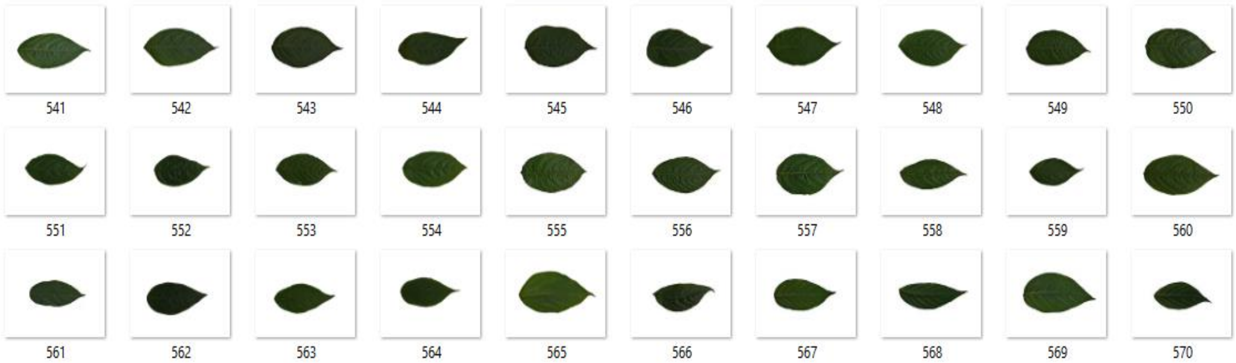

Supplement: Supplemental Information 1 — Thumbnail view of myDAUN dataset. [file peerj-05-3792-s001.pdf]
